# The Role of Quantitative Ultrasound in Monitoring Neoadjuvant Chemotherapy in Breast Cancer: A Narrative Review

**DOI:** 10.3390/cancers17223676

**Published:** 2025-11-17

**Authors:** Hanna Piotrzkowska-Wróblewska

**Affiliations:** Ultrasound Department, Institute of Fundamental Technological Research, Polish Academy of Sciences, 02-106 Warsaw, Poland; hpiotrzk@ippt.pan.pl

**Keywords:** breast cancer, neoadjuvant chemotherapy, quantitative ultrasound (QUS), treatment response monitoring

## Abstract

Breast cancer remains the most commonly diagnosed malignancy and a leading cause of cancer-related mortality among women worldwide. Neoadjuvant chemotherapy (NAC) is increasingly used, particularly in aggressive subtypes such as HER2-positive and triple-negative breast cancer, where achieving a pathological complete response (pCR) is strongly associated with improved outcomes. Early and accurate assessment of therapeutic response is therefore essential to enable timely treatment adaptation. Conventional imaging methods—including magnetic resonance imaging (MRI), computed tomography (CT), mammography, and B-mode ultrasound—mainly detect macroscopic tumor shrinkage and often lagging behind biological alterations, as they rely primarily on size-based assessment. Quantitative ultrasound (QUS) is an emerging, non-invasive technique that analyzes raw radiofrequency (RF) ultrasound data to extract spectral, scattering, and attenuation parameters, allowing detailed characterization of tumor microstructure. When combined with parametric mapping, texture analysis, and advanced radiomic or deep learning approaches, QUS can capture subtle tissue alterations at an early stage of therapy and help predict pathological response before conventional imaging detects morphologic change. Integration with molecular and clinical data further enhances predictive performance, enabling adaptive and personalized treatment strategies. This narrative review summarizes current evidence on the clinical utility of QUS in monitoring NAC response in breast cancer, outlines the methodological foundations of this technology, and discusses key challenges to its broader implementation—particularly the need for standardized acquisition and processing protocols, robust interpretive algorithms and large, prospective, multicenter validations to confirm its impact on clinical decision-making and patient outcomes, and to accelerate its translation into precision oncology practice.

## Simple Summary

Breast cancer remains the most frequently diagnosed malignancy among women worldwide, with rising incidence and significant biological heterogeneity influencing treatment strategies. Neoadjuvant chemotherapy (NAC) has become a standard option, particularly for aggressive molecular subtypes, underscoring the need for sensitive tools to monitor early treatment response. Conventional imaging (MRI, CT, mammography, and B-mode ultrasound) primarily captures morphological change, often lagging biological alterations. Quantitative ultrasound (QUS) is an emerging modality that characterizes tumor microstructure and yields reproducible, operator-independent biomarkers. This narrative review synthesizes current evidence, clarifies the conceptual framework (spectral, amplitude, and attenuation metrics; parametric maps and texture), highlights clinical applications and limitations, and outlines future directions for integrating QUS into NAC response assessment in breast cancer.

## 1. Introduction

Breast cancer remains one of the most significant oncologic challenges worldwide and is the most frequently diagnosed malignancy among women. In 2020, over 2.3 million new cases and nearly 685,000 deaths were recorded, accounting for 11.7% of all cancer diagnoses and 6.9% of cancer-related mortality [1]. Despite advances in screening and treatment, survival disparities persist, particularly in regions with limited access to modern diagnostics and therapies [2]. In the United States, breast cancer is the most commonly diagnosed cancer in women and the second leading cause of cancer-related death [3,4], with similar trends observed across Europe and North America [2,5,6].

Rising incidence among younger women and pronounced biological heterogeneity pose ongoing challenges for diagnosis and treatment [7,8,9]. Molecular diversity influences prognosis and therapeutic response, underscoring the need for individualized approaches and tools capable of detecting early biological changes during therapy [1,2,4,5,7,9,10,11]. Four principal molecular subtypes—luminal A, luminal B, HER2-enriched, and triple-negative breast cancer (TNBC)—differ in receptor expression and proliferation markers, which directly influence prognosis and treatment strategies [12,13,14,15,16,17].

These subtype-specific characteristics guide eligibility for neoadjuvant chemotherapy (NAC). In aggressive tumors such as HER2-positive and TNBC, NAC frequently induces pathological complete response (pCR), strongly associated with improved survival [15,17,18,19,20,21]. NAC also reduces tumor volume, increases breast-conserving surgery rates, optimizes axillary management, and enables real-time assessment of tumor chemosensitivity [22,23,24,25,26]. As NAC use expands to biologically aggressive early-stage tumors, the need for reliable early-response assessment becomes increasingly important [27,28,29,30,31]. Current response evaluation relies largely on morphologic measurements such as RECIST [32], which do not capture microstructural changes that precede reduction in tumor size [27,28].

Multiple imaging modalities—MRI, CT, B-mode ultrasound, and mammography—are currently used for NAC monitoring. MRI offers the highest diagnostic accuracy but remains limited by cost, accessibility, and reliance on morphology. CT is widely available but less sensitive, while B-mode ultrasound is inexpensive yet operator-dependent. Mammography has limited utility in dense breasts. A concise comparison of these modalities is presented in Table 1 [27,28,33,34,35].

Taken together, currently available imaging approaches lack sufficient sensitivity to detect early biological alterations during therapy—changes that often precede measurable tumor shrinkage. This gap is particularly critical for aggressive subtypes such as HER2-positive and TNBC, where timely identification of non-responders may enable rapid treatment modification.

Against this background, quantitative ultrasound (QUS) has emerged as a promising noninvasive technique for early response assessment. Unlike conventional B-mode imaging, which relies on qualitative echogenicity, QUS analyzes raw radiofrequency (RF) data to extract spectral and scattering parameters reflecting tissue microstructure [36,37,38,39,40]. This enables detection of therapy-induced changes before morphological effects become apparent [36,37,38]. Integrating QUS with texture analysis, radiomics, and molecular information further enhances predictive performance, with studies demonstrating the ability of QUS-based models to localize resistant tumor regions and predict NAC response early during treatment [39,40,41,42,43,44,45,46]. Such approaches may enable more personalized and adaptive therapeutic strategies.

This narrative review summarizes peer-reviewed literature (2010–2025) identified through PubMed, Web of Science, and Google Scholar on quantitative ultrasound (QUS) applications in monitoring breast cancer response to neoadjuvant chemotherapy.

## 2. Quantitative Ultrasound: Physical Principles and Biological Basis for Cancer Therapy Monitoring

Quantitative ultrasound relies on the analysis of raw, uncompressed radiofrequency (RF) echoes backscattered from internal tissue structures and recorded by the ultrasound transducer before beamforming and log-compression. RF data preserve both the amplitude and phase of the ultrasonic wave over time, enabling advanced quantitative analyses [47,48,49]. Although RF signals are produced by most ultrasound systems, clinical access is not routine, which limits widespread adoption and often requires research modes or systems that allow raw RF export [47,48]. For reproducible biomarkers, QUS pipelines should include reference-phantom calibration and attenuation compensation, reported alongside acquisition settings [47,48,49].

The fundamental principle of quantitative ultrasound (QUS) is that the microstructure of biological tissue—defined by the spatial distribution and acoustic properties of cells, nuclei, collagen fibers, and microvasculature—directly affects the scattering and attenuation of ultrasonic waves. Quantitative analysis of these effects can be performed in the frequency domain, through spectral analysis yielding parameters such as the midband fit (MBF), spectral slope (SS), and 0 MHz intercept (SI), as well as scatterer-based metrics including the effective scatterer diameter (ESD) and effective acoustic concentration (EAC). Alternatively, it can be conducted in the amplitude domain using statistical models such as the Rayleigh, Nakagami, or homodyned-K distributions [50,51,52,53,54].

In oncology, QUS provides a particularly valuable framework for monitoring therapy-induced microstructural alterations. Within tumor tissue, the earliest changes following neoadjuvant chemotherapy (NAC) include cellular reorganization and modifications in the architecture and density of the extracellular matrix, which may occur within the first few days of treatment and reflect an initial cytotoxic response [55]. As therapy progresses, nuclear fragmentation and the formation of apoptotic bodies emerge as morphological hallmarks of active cell death, while continued treatment leads to the development of necrotic regions that signify cumulative structural degradation [56]. In parallel, NAC remodels the tumor microenvironment by altering fibroblast populations, lymphocytic infiltration, and epithelial-to-mesenchymal transition (EMT)–related gene expression, processes that can influence subsequent treatment sensitivity and overall prognosis [55,56]. These biological events typically precede measurable tumor shrinkage on conventional imaging, emphasizing the unique value of quantitative and reproducible QUS biomarkers for the early assessment of therapeutic efficacy [55,56,57].

QUS is therefore uniquely suited to capture these subtle microarchitectural transformations. Spectral parameters such as MBF, SS, and SI, together with scatterer metrics like ESD and EAC, demonstrate stage-specific correlations with biological response to therapy. For example, early increases in MBF and SS have been associated with nuclear fragmentation and apoptosis, while changes in ESD and EAC are linked to necrosis and extracellular-matrix reorganization [40,50,58,59,60,61]. Clinically, significant alterations in these QUS parameters are often detectable within the first weeks of NAC in patients who respond favorably to treatment, whereas in non-responders such changes are minimal or absent [58,59,60,61].

These properties make QUS a powerful tool for early therapy monitoring, especially in biologically aggressive subtypes-characterized by rapid progression and a high risk of recurrence-early identification of non-responders is critical for timely treatment modification. Achieving a pathological complete response (pCR) substantially improves prognosis, whereas the absence of pCR is associated with increased recurrence and poorer survival [62,63]. Early, quantitative assessment using QUS may therefore guide personalized decisions, allowing clinicians to adapt or intensify therapy and ultimately improve outcomes. The following section details key QUS parameters and their biophysical relationships with tissue microstructure, which form the basis of their diagnostic and prognostic value in breast cancer therapy monitoring.

## 3. QUS Parameters and Their Significance in Assessing Tissue Microstructure

### 3.1. Spectral QUS Parameters

Spectral analysis of the ultrasound signal is a key component of quantitative ultrasound (QUS), enabling assessment of tissue microstructure from the frequency content of the backscattered radiofrequency (RF) signal. Unlike conventional amplitude analysis, which primarily reflects backscatter intensity, spectral analysis conveys information about the size, shape, and spatial organization of acoustic scatterers such as cells, nuclei, and collagen fibers [64,65,66]. This stems from the relation between frequency and scatterer scale—higher frequencies interact preferentially with smaller structures, whereas lower frequencies are more sensitive to larger ones. Consequently, the spectrum serves as an indirect descriptor of dominant structural dimensions, allowing sub-microscopic characterization that is relevant both for lesion differentiation and therapy monitoring [65,66,67].

A major advantage of spectral parameters is their repeatability and limited dependence on scanner presets or operator technique, as they are computed from raw RF data. Among primary features, the spectral slope (SS) estimates predominant scatterer size by the slope of a linear regression fitted to the log-power spectrum within a defined band (steeper, more negative slopes → finer structures) [64,65,66]. The 0 MHz intercept (SI) is the extrapolated spectrum value near zero frequency, reflecting overall backscatter strength [64,65]. The midband fit (MBF) is the mean value of the fitted regression line within the mid-band of the analyzed spectrum and captures information related to scatterer density/organization [65,66,67].

In practice, spectral features are derived through standardized QUS pipelines involving reference-phantom calibration and attenuation correction, ensuring reproducibility across systems and acquisition settings. Growing interest also targets model-based spectral parameters, which provide deeper biophysical insight. Particularly important are the backscatter coefficient (BSC) and quantities derived from scattering models, including the effective scatterer diameter (ESD) and effective acoustic concentration (EAC)—log-scaled density of scatterers; their estimation typically adopts Rayleigh, Gaussian, or related form-factor models [64,66]. These descriptors are valuable in translational and experimental studies, where precise microstructural characterization underpins diagnostic and prognostic evaluation.

The clinical value of spectral parameters has been confirmed in numerous studies demonstrating their ability to detect early microstructural changes in response to neoadjuvant chemotherapy [32,65,66,67,68]. Furthermore, spectral parameters constitute the foundation for radiomics and AI pipelines. Multiparametric QUS features integrated with molecular and clinical data can improve response prediction and enable personalized treatment planning. Through these properties, spectral analysis forms a central pillar of strategies for early, non-invasive monitoring of cancer therapy efficacy.

A concise summary of key spectral parameters is provided in Table 2.

Spectral QUS parameters provide quantitative insight into the microstructural organization of tumor tissue. The spectral slope (SS) primarily reflects the dominant scatterer size, the 0 MHz intercept (SI) corresponds to the overall backscatter strength, and the midband fit (MBF) represents the mean backscatter level within the analyzed frequency range. Model-based parameters such as the effective scatterer diameter (ESD) and effective acoustic concentration (EAC) further quantify scatterer size and density. Clinically, early increases in MBF or SS have been associated with apoptosis and microstructural disorganization during effective therapy, whereas stable or decreasing values may indicate therapeutic resistance. Together, these parameters form the foundation for reproducible, biologically interpretable biomarkers of treatment response and provide the quantitative basis for radiomic and machine-learning models in oncologic imaging.

### 3.2. Amplitude-Based QUS Parameters

Amplitude-based quantitative ultrasound (QUS) complements spectral analysis by modeling the statistical distribution of RF-envelope amplitudes within a region of interest, which enables quantitative characterization of tissue heterogeneity, organization, and microarchitectural complexity [48,69,70]. The most widely used probability laws are Rayleigh, Nakagami, and homodyned-K, which link the shape of the amplitude histogram to the density and spatial arrangement of scatterers [48,69,70,71,72,73]. The Nakagami parameter (m) is informative for homogeneity: m ≈ 1 corresponds to Rayleigh (fully developed speckle), m < 1 to more heterogeneous/sparse scattering, and m > 1 to increasingly coherent/ordered microstructure [48,69,70]. The homodyned-K distribution flexibly spans conditions from Rayleigh-like speckle to signals dominated by strong, discrete scatterers, as may occur when proliferating tumor cells coexist with fibrous or calcified components [48,71,72,73].

In practice, these amplitude-based features are derived from envelope statistics after demodulation of raw RF data and are corrected for acquisition settings and depth-dependent attenuation to ensure comparability across systems. In addition, to classical probabilistic models, several complementary parameters enhance the ability of QUS to quantify complex tissue structure. Entropy of the envelope quantifies disorder in the backscatter pattern, while Kullback–Leibler (KL) divergence measures deviation of the tumor’s amplitude distribution from that of a reference tissue (e.g., contralateral or peritumoral) [48,70,74,75]. Elevated entropy or KL divergence indicate chaotic, heterogeneous architecture; their progressive decline during therapy can reflect homogenization or acoustic normalization [48,70,74,75].

The effective number of scatterers (ENS) estimates the effective scatterer population and typically decreases with necrosis or structural homogenization, whereas generalized-gamma shape parameters (α, β) adapt to real histograms and sensitively capture microstructural reorganization [48,69,70,72]. Homodyned-K moments further characterize composite signals generated when small cellular scatterers coexist with larger fibrous or calcified inclusions—features common in solid tumors under treatment [48,71,73].

Clinically, amplitude-based parameters complement spectral QUS by adding information on heterogeneity and organization of the tumor microenvironment [48,69,70,74]. Multiple studies report early on-treatment changes—within the first weeks of neoadjuvant chemotherapy-in Nakagami—m, entropy/ENS, and homodyned-K-based descriptors that separate responders from non-responders before measurable size reductions occur (Table 3) [48,70,74,75].

Amplitude-based QUS parameters extend spectral analysis by quantifying tumor heterogeneity, organization, and tissue remodeling during therapy. The Nakagami parameter (m) and homodyned-K statistics describe scatterer clustering and coherence, while entropy and KL divergence reflect structural disorder relative to normal tissue. Measures such as ENS or generalized-gamma coefficients capture reductions in effective scatterer number and evolving microstructural uniformity associated with apoptosis, necrosis, and fibrosis. Clinically, decreases in heterogeneity metrics and normalization of amplitude distributions have been linked with favorable therapeutic response, whereas persistent disorder may indicate treatment resistance. In combination with spectral and attenuation parameters, amplitude-based QUS offers a robust and biologically interpretable framework for monitoring neoadjuvant chemotherapy response.

### 3.3. Attenuation Coefficient (AC)

Among the quantitative ultrasound (QUS) parameters, the attenuation coefficient (AC) plays a particularly important role, as it reflects the decrease in the amplitude of the ultrasonic signal as it propagates through tissue. This phenomenon results from the combined effects of energy absorption and scattering by internal tissue structures. AC values depend on the physical and microstructural properties of the examined tissue, including cell density, extracellular matrix composition, collagen content, and vascularization [82,83,84,85,86]. Consequently, the attenuation coefficient can serve as an indirect biomarker of biological changes occurring within tumors during systemic therapy.

Measurement of AC is based on analyzing the relationship between the amplitude (or energy) of the radiofrequency (RF) signal and depth of penetration. Techniques include spectral comparison with reference media, energy-ratio algorithms across frequency bands, and more advanced methods such as wavelet-domain and model-based estimation [84,85]. Because soft tissues naturally attenuate ultrasound waves, accurate estimation of AC requires correction for propagation losses and careful control of acquisition parameters. A major limitation in clinical research remains the lack of standardized acquisition and processing protocols, which hampers direct comparison of results across institutions [85,86].

To address this challenge, international standardization efforts, such as those led by the Quantitative Imaging Biomarkers Alliance (QIBA) of the Radiological Society of North America (RSNA), and by the International Electrotechnical Commission (IEC), are developing consensus guidelines and reference phantoms to harmonize acquisition parameters and analysis pipelines for attenuation imaging. These initiatives aim to improve reproducibility and facilitate the clinical translation of AC-based QUS techniques [85,86].

In the context of monitoring the response to neoadjuvant chemotherapy (NAC) in breast cancer, AC provides valuable insight into microstructural remodeling of tumor tissue. Effective treatment triggers a cascade of biological processes—including cellular reorganization, nuclear fragmentation, formation of apoptotic bodies, and subsequent necrosis and fibrosis—that significantly alter tissue acoustic properties. Clinical studies have shown that, in well-responding patients, AC exhibits measurable and reproducible changes after only a few treatment cycles. Several studies have reported an increase in AC, attributed to the development of fibrosis and necrosis, which enhance absorption and scattering of ultrasound waves. Others have described a decrease in AC, interpreted as a result of cell disintegration and tissue homogenization. Conversely, a lack of significant AC variation has been consistently observed in non-responding tumors and may serve as an early indicator for treatment adaptation or intensification [87,88,89].

From a clinical standpoint, interpreting AC requires caution because the direction and magnitude of its change can depend on multiple factors, including ultrasound frequency, treatment regimen, analyzed tissue region (e.g., tumor core vs. peritumoral margin), and vascular status. Nevertheless, dynamic AC monitoring during NAC remains a promising, non-invasive adjunct to conventional morphological imaging, providing early insight into treatment efficacy and complementing other QUS biomarkers.

### 3.4. Parametric Mapping and Texture-Based Assessment of Tumor Heterogeneity

The mean values of QUS parameters provide information on the global acoustic properties of the tumor; however, their spatial analysis in the form of parametric maps allows for capturing the internal heterogeneity of its microstructure [90,91]. Parametric maps depict the spatial distribution of locally calculated parameter values across the entire tumor, enabling visualization of regions exhibiting varying degrees of biological response. The process of generating such maps involves the acquisition of raw RF data, segmentation of the region of interest, computation of parameters within a sliding analysis window, and interpolation of the results to a spatial grid. The spatial resolution of the maps depends on the size of the analysis window: smaller windows allow for the detection of fine microarchitectural details but increase noise levels, whereas larger windows yield more stable estimates at the cost of losing information about small-scale structures.

A natural extension of the parametric mapping approach is texture analysis, which describes the spatial organization of signals within QUS maps [92]. Classical texture analysis utilizes parameters derived from the gray-level co-occurrence matrix (GLCM), such as entropy, contrast, homogeneity, and correlation [93]. These parameters reflect subtle differences in tissue architecture—for example, an increase in entropy indicates higher tumor heterogeneity, a decrease in contrast and correlation reflects loss of structural organization, and variations in homogeneity may suggest remodeling of the extracellular matrix fibers [94,95].

In recent years, higher-order matrices have been increasingly applied to capture more complex spatial patterns. Examples include the gray-level run-length matrix (GLRLM) and the gray-level difference matrix (GLDM), which describe the length and intensity of sequences of similar pixel values. The gray-level amplitude matrix (GLAM) enables quantitative assessment of amplitude variations relative to gray-level intensity, providing additional information on the spatial dynamics of ultrasonic energy distribution [90,96,97]. Parameters such as short-run emphasis, long-run emphasis, and high gray-level emphasis have proven particularly useful in describing the degree of tissue organization and its remodeling in response to therapy. Even more advanced methods include higher-order statistical analyses, such as third- and fourth-order moments (skewness and kurtosis of texture distributions), and wavelet-based radiomics, which decompose parametric maps into frequency components and analyze spatial patterns at multiple scales. Such “texture derivatives,” as described by Dasgupta et al. extend the classical QUS radiomic framework and enable the development of more complex predictive indices [97]. In their study on QUS radiomics for evaluating response to neoadjuvant chemotherapy (NAC) in patients with locally advanced breast cancer, the inclusion of higher-order texture features significantly improved predictive accuracy compared to first-order metrics.

From a clinical perspective, the relevance of QUS texture analysis is twofold. First, it enables the detection of subtle, regional variations in tumor response that may reflect intratumoral heterogeneity, including the presence of subpopulations of cells with varying sensitivity to treatment. Second, aggregation of information from texture maps—for instance, the proportion of high-entropy or low-homogeneity regions within the entire tumor—may serve as a predictive biomarker at the individual patient level. Thus, texture analysis of QUS parametric maps not only complements traditional morphological assessments but also substantially enhances the potential for therapy personalization and clinical decision support during neoadjuvant treatment.

**Figure 1:**
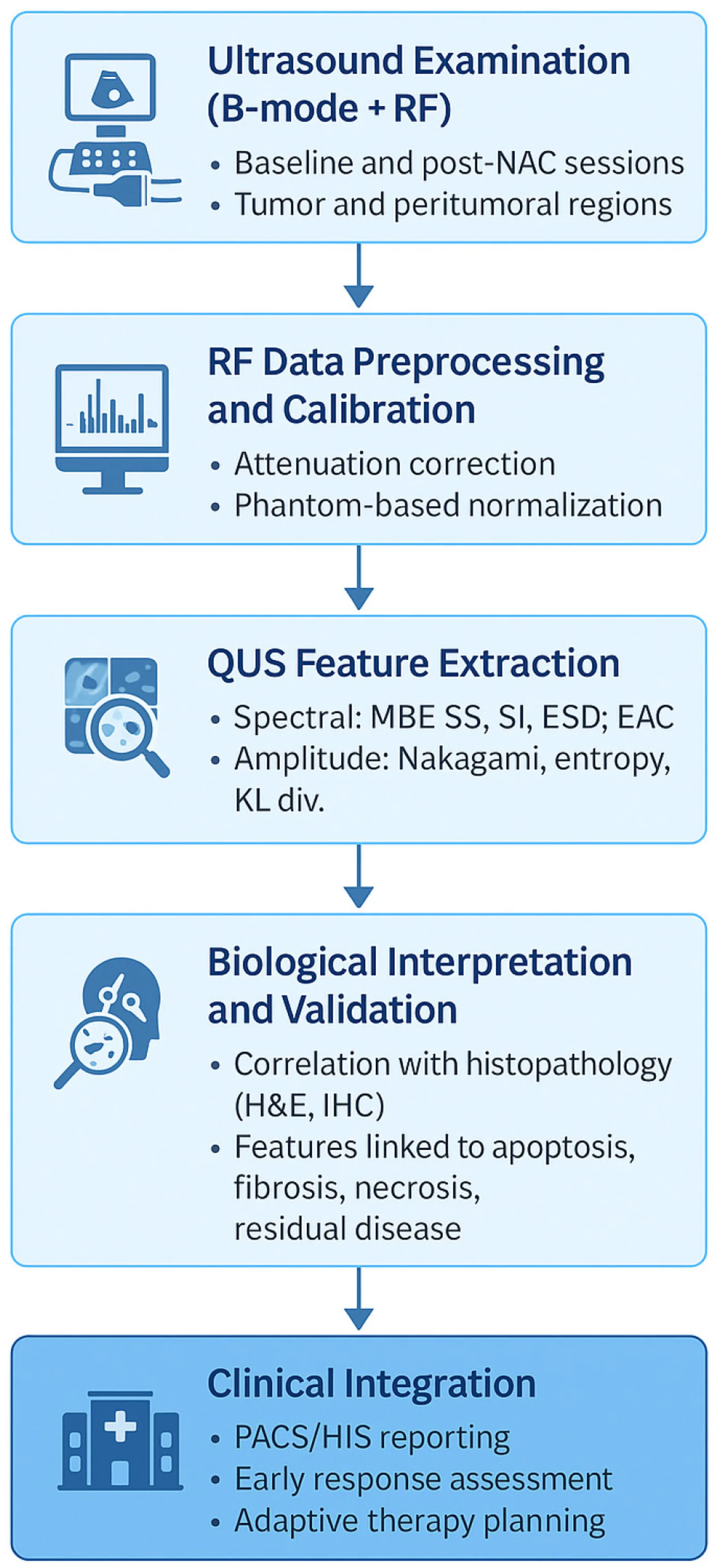
Workflow of quantitative ultrasound (QUS) for monitoring neoadjuvant chemotherapy (NAC) response in breast cancer. The diagram summarizes the analytical and translational pipeline: acquisition of radiofrequency (RF) data, preprocessing and calibration, extraction of spectral, amplitude-based, and attenuation parameters, generation of parametric and texture maps, correlation with histopathologic findings (H&E, IHC), and integration into clinical decision-support systems (PACS/HIS). QUS biomarkers capture microstructural remodeling such as apoptosis, necrosis, and fibrosis, enabling early response assessment and adaptive treatment planning.

A schematic overview of the quantitative ultrasound (QUS) workflow, illustrating the analytical pipeline from raw RF data to clinical interpretation and histologic correlation, is presented in Figure 1.

### 3.5. Integration of QUS with Other Data Sources

Integration of quantitative ultrasound (QUS) with clinical, molecular, and multimodal imaging data represents one of the key directions in advancing methods for monitoring response to neoadjuvant chemotherapy (NAC) in breast cancer. Incorporating QUS biomarkers—based on parameters such as scattering, attenuation, and amplitude-based signal statistics—together with clinical factors (e.g., age, tumor stage, lymph node status, Ki-67 expression), molecular characteristics (e.g., ER, PR, HER2 status, molecular subtypes, transcriptomic signatures), and additional imaging modalities (MRI, mammography, elastography), as well as radiomic features and artificial intelligence (AI) algorithms, enables the construction of multimodal predictive models with high translational potential [38,39,40,41,42,47,48,53,98,99,100,101,102].

This integrative approach reflects a broader trend in modern oncologic imaging, where combining multiple complementary modalities and data sources allows for a more comprehensive understanding of tumor biology and treatment response. By linking clinical, molecular, and imaging information, such models provide a multidimensional framework that enhances both predictive accuracy and biological interpretability.

A particularly valuable aspect of this integration is the ability to capture and visualize tumor heterogeneity. QUS parametric maps can be transformed into regional probability maps of treatment response, enabling identification of subregions with differential chemosensitivity and supporting biopsy targeting or surgical margin planning [7,99,102]. These spatially resolved biomarkers bridge the gap between quantitative imaging and therapeutic decision-making, offering a unique opportunity for precision-guided interventions.

Current modeling strategies employ both conventional machine learning algorithms—such as support vector machines (SVM), k-nearest neighbors (KNN), and linear discriminant analysis (LDA)—and advanced deep learning architectures, including convolutional neural networks (CNN), 3D-CNNs, and vision transformers (ViT). These models are capable of analyzing complex spatiotemporal heterogeneity patterns within volumetric QUS data [42,46,90,98,99,100,101,102]. Successful implementation of such approaches requires harmonization of imaging data across centers, control of confounding variables, and the incorporation of explainable artificial intelligence (XAI) methods to ensure transparency, reproducibility, and clinical interpretability of predictions.

Together, these developments highlight the growing role of QUS as a core component of multimodal imaging and integrative oncology, providing quantitative, biologically grounded biomarkers that can guide individualized therapy and support the transition toward adaptive, data-driven cancer care.

## 4. Translational and Clinical Applications of Quantitative Ultrasound in Breast Cancer

Over the past two decades, quantitative ultrasound (QUS) has evolved from a research-oriented imaging technique into a promising biomarker-driven clinical tool for characterizing breast tumor microstructure, monitoring therapeutic response, and supporting personalized treatment strategies. The translational pathway of QUS—from biophysical validation in phantoms and animal models, through single-center clinical studies, to ongoing multi-institutional AI-integrated trials—illustrates its growing maturity and readiness for clinical translation.

This section provides a narrative synthesis of key evidence across preclinical and clinical contexts, highlighting how QUS-derived biomarkers contribute to early therapy-response prediction, biological interpretation of tissue remodeling, and adaptive refinement of neoadjuvant chemotherapy regimens

### 4.1. Preclinical Studies (Mice, Phantoms, In Vitro Models)

The foundations of quantitative ultrasound (QUS) as a reliable diagnostic and prognostic imaging modality were established through preclinical research. Experimental studies using animal models, acoustic phantoms, and in vitro systems provided essential insights into how ultrasonic backscatter relates to tissue microstructure and therapy-induced biological alterations. These controlled environments allowed for systematic evaluation of QUS parameters, analysis algorithms, and acquisition protocols before their translation into clinical practice.

Among these approaches, murine xenograft models play a central role, enabling longitudinal and non-invasive monitoring of microstructural changes during chemotherapy, radiotherapy, or targeted therapy. In pioneering work, Czarnota and colleagues demonstrated that high-frequency ultrasound can detect apoptosis in vitro, in situ, and in vivo, with characteristic spectral and backscatter changes corresponding to chromatin condensation and nuclear fragmentation [103]. Subsequent studies confirmed that QUS-derived parameters—such as midband fit (MBF), spectral slope (SS), 0 MHz intercept (SI), and scattering metrics including the effective scatterer diameter (ESD) and effective acoustic concentration (EAC)—correlate strongly with biological processes such as apoptosis, extracellular matrix remodeling, and changes in cellular density [36,40,58,104]. These biomarkers have been consistently validated through histopathology and immunohistochemical assays of cell death, reinforcing their translational significance.

Acoustic phantoms, designed to mimic the scattering and attenuation properties of soft tissues, constitute an essential calibration tool in preclinical QUS research. They enable precise control of scatterer number, size, and spatial distribution, facilitating systematic assessment of how these factors influence QUS spectral and statistical parameters [47,48]. By providing standardized reference media, phantom-based calibration enhances reproducibility and ensures cross-platform comparability of QUS measurements across experimental setups and institutions.

In vitro models complement these approaches by offering full control over the microarchitecture of the studied material. Engineered tissue constructs and cultured cell systems allow isolation of specific structural features—such as cell size, packing density, and extracellular composition—and quantitative correlation of these characteristics with radiofrequency (RF) signal statistics. Such experiments have been instrumental in identifying QUS parameters most sensitive to distinct biological processes, including apoptosis, necrosis, and fibrosis [47,48,58,103].

Collectively, preclinical investigations have established the biophysical foundations and translational relevance of QUS biomarkers, demonstrating their sensitivity to cell death, extracellular matrix reorganization, and therapy-induced microstructural remodeling. This evidence provides the mechanistic rationale for subsequent clinical investigations in breast cancer and other solid tumors.

A summary of the principal preclinical QUS applications across animal, phantom, and in vitro models is presented in Table 4.

### 4.2. Single-Center Clinical Studies of QUS

Following the promising results obtained in preclinical tumor models, quantitative ultrasound (QUS) was introduced into clinical studies involving patients with breast cancer undergoing neoadjuvant chemotherapy (NAC). Early observations demonstrated that spectral parameters such as midband fit (MBF), spectral slope (SS), and 0 Mhz intercept (SI) increased within the first 1–2 weeks of therapy in patients who responded favorably to treatment. These changes were interpreted as signatures of apoptosis and microarchitectural remodeling of tumor tissue [106]. Subsequent analyses confirmed that early alterations in these parameters correlated with both NAC response and long-term outcomes [40,58,107].

Expanded single-center studies investigated tumor heterogeneity and texture within QUS parametric maps. Combining conventional QUS parameters with textural and molecular features enabled early prediction of treatment response with accuracies of 78–86% during the initial therapy phase [41]. Another study assessed the repeatability of QUS biomarkers, confirming high inter-system consistency under clinical conditions [108].

A major advancement was the demonstration that baseline QUS parameters—obtained before NAC initiation and measured in both tumor core and peritumoral regions—could predict treatment efficacy and overall survival, highlighting the potential of QUS for early patient stratification [36]. Building on this, radiomic analyses incorporating higher-order texture derivatives further improved predictive performance, enabling earlier differentiation between pathologic complete responders (pCR) and non-responders [97].

Further investigations confirmed the utility of statistical scattering parameters and the integrated backscatter coefficient (IBSC) for early response assessment, yielding AUC values up to 0.91 when combining multiple QUS biomarkers, and approximately 0.82 after the second or third NAC cycle for IBSC alone [109]. Quantitative echogenicity analyses also revealed significant associations with treatment outcomes, allowing early differentiation between responders and non-responders [74]. Moreover, divergence metrics derived from RF-envelope signal distributions—such as Kullback–Leibler and Kolmogorov–Smirnov statistics—enabled detection of non-response as early as after the first NAC dose (AUC ≈ 0.83–0.84), with predictive accuracy improving to ≈0.90–0.91 after the third dose [110].

Significant progress has also resulted from the integration of machine-learning and deep-learning techniques. Combining QUS biomarkers with radiomic features and employing transfer learning on parametric maps improved prediction accuracy even before therapy initiation [42,46,100]. Furthermore, early QUS parameter changes—detectable as early as week 4 of therapy—were associated not only with pathologic response but also with recurrence risk, underscoring their prognostic value [90]. Most recently, prospective validation studies have confirmed the clinical utility of QUS-based models, including textural biomarkers, and their potential role in guiding early treatment intensification during NAC [111].

To provide a comprehensive overview of this evidence, Table 5 summarizes the principal single-center clinical studies employing QUS for monitoring breast cancer response to neoadjuvant chemotherapy.

**Figure 2:**
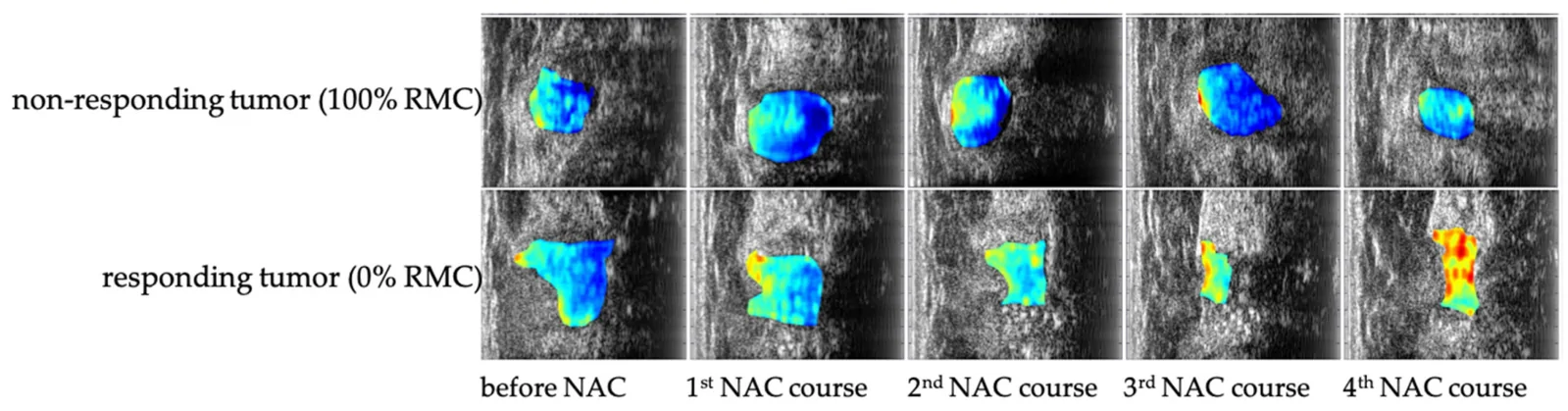
Representative quantitative ultrasound (QUS) parametric maps based on the integrated backscatter coefficient (IBSC) overlaid on B-mode images of breast tumors during neoadjuvant chemotherapy (NAC). The upper row shows IBSC parametric maps from a patient exhibiting no response to NAC, characterized by persistently low and spatially homogeneous backscatter values.

To complement the findings summarized in Table 5, Figure 2 presents representative examples of quantitative ultrasound (QUS) parametric maps overlaid on B-mode images of breast tumors obtained during neoadjuvant chemotherapy. The integrated backscatter coefficient (IBSC) maps visualize spatial heterogeneity and temporal microstructural changes associated with treatment response. Warmer color regions (yellow–red) correspond to areas of increased backscatter intensity and structural organization, while cooler regions (blue–cyan) indicate decreased scattering and tissue homogenization. These examples illustrate how QUS biomarkers such as IBSC enable non-invasive visualization of therapy-induced remodeling, supporting early differentiation between responding and non-responding tumors.

The lower row presents images from a patient achieving a pathologic complete response (0% RMC—Residual Malignant Cells), with marked increases in IBSC and pronounced spatial heterogeneity reflecting therapy-induced microstructural remodeling. Warmer colors (red–yellow) indicate regions of increased scattering and tissue organization, whereas cooler tones (blue–cyan) correspond to homogenized or necrotic tissue areas. Together, these examples illustrate the potential of QUS biomarkers to non-invasively visualize treatment response dynamics during NAC.

### 4.3. Integration of Quantitative Ultrasound with Multimodal and Multisource Data

The expansion of quantitative ultrasound (QUS) beyond traditional single-parameter analysis toward the integration of imaging, molecular, and clinical data marks a new stage in the evolution of methods for monitoring response to neoadjuvant chemotherapy (NAC) in breast cancer. Combining QUS biomarkers—based on parameters such as scattering, attenuation, and amplitude-based signal statistics—with clinical factors (e.g., patient age, tumor stage, lymph node status, Ki-67 expression), molecular characteristics (e.g., ER, PR, HER2 status, molecular subtypes, transcriptomic signatures), and other imaging modalities (MRI, mammography, elastography), as well as radiomic features and artificial intelligence (AI) algorithms, enables the construction of multimodal predictive models with high translational potential [38,39,40,41,42,47,48,53,98,99,100,101,102].

A particularly valuable aspect of such integration is its ability to capture and visualize intratumoral heterogeneity. QUS parametric maps can be transformed into spatial probability maps of treatment response, allowing identification of subregions with differential chemosensitivity and supporting targeted biopsy planning or surgical margin assessment [7,99,102]. This visual representation of biological variability within the tumor provides a powerful framework for personalizing therapy and adapting treatment strategies in real time.

Modern modeling strategies employ both traditional machine-learning algorithms—such as support vector machines (SVM), k-nearest neighbors (KNN), and linear discriminant analysis (LDA)—and advanced deep-learning architectures, including convolutional neural networks (CNN), three-dimensional CNNs (3D-CNN), and vision transformers (ViT). These models are capable of capturing complex spatiotemporal patterns in volumetric QUS data [42,46,90,98,99,100,101,102]. Successful implementation requires harmonization of imaging data across centers and platforms, control of confounding factors, and the incorporation of explainable artificial intelligence (XAI) frameworks to ensure transparency and interpretability of predictions for clinical decision-making.

Integrated approaches that combine QUS data with clinical, molecular, and radiomic information significantly enhance the accuracy and robustness of treatment-response prediction. Such models enable early therapeutic adaptation and support precision-oncology strategies. Preliminary validations have confirmed their reproducibility and reliability, underscoring their translational potential and clinical relevance in modern oncology [38,41,42,45,46,99,100,101,102,113,114,115,116,117,118,119].

The most representative studies illustrating these integrative approaches—combining QUS with clinical, molecular, and radiomic data—are summarized in Table 6, which outlines the chronological evolution from early spectral–texture models to advanced deep-learning and multimodal frameworks.

### 4.4. Multi-Institutional Validation of QUS for Monitoring Neoadjuvant Chemotherapy Response

The development of multi-institutional studies marks a crucial step in translating quantitative ultrasound (QUS) from experimental and single-center research into clinical application. These collaborative efforts have enabled evaluation not only of acquisition reproducibility and inter-system comparability of QUS parameters but also of the robustness of predictive models across diverse patient populations and real-world clinical settings [100,101].

Large-scale international collaborations have contributed to the standardization of QUS acquisition and analysis protocols, reducing operator- and equipment-dependent variability. As a result, QUS has been demonstrated to be a reproducible and reliable imaging biomarker for monitoring neoadjuvant chemotherapy (NAC) response across multiple centers, with high predictive consistency and cross-platform generalizability [100,101].

Table 7 summarizes the key multi-institutional studies evaluating the clinical performance of QUS in NAC response monitoring. One prospective investigation conducted across four centers showed that radiomic features extracted from pre-treatment QUS data predicted NAC response with an accuracy of 87%, supporting early patient stratification and treatment personalization [100]. Another multi-center study involving three institutions found that in-treatment QUS features acquired at weeks 1 and 4 outperformed baseline parameters in predicting therapeutic response, achieving an area under the curve (AUC) of 0.87 using a support vector machine classifier [100]. Complementary findings from a separate multi-institutional cohort demonstrated that combining QUS, texture, and molecular features enabled early prediction of response with an accuracy of up to 86% at week 4 [39].

A major translational milestone was achieved through a randomized, multi-institutional feasibility trial implementing QUS-guided adaptive NAC. In this study, early prediction of therapeutic response enabled real-time treatment modification and was successfully integrated into clinical workflows, demonstrating feasibility for precision oncology applications [101].

Building on these efforts, recent deep learning–based approaches have been applied to longitudinal ultrasound datasets collected from multiple hospitals. A Siamese multi-task neural network trained on such data achieved AUC values ranging from 0.90 to 0.99 in external validation, confirming the potential of advanced QUS analytics for early and accurate prediction of pathological response [121].

Together, these multi-institutional studies highlight the maturity of QUS as a translational imaging biomarker and underscore the importance of standardized, large-scale validation to enable its eventual clinical adoption for adaptive therapy monitoring.

## 5. Limitations and Future Directions of Quantitative Ultrasound in Neoadjuvant Therapy Monitoring

### 5.1. Clinical Outlook and Early Predictive Potential

The principal clinical advantage of quantitative ultrasound (QUS) in the neoadjuvant setting lies in its exceptional sensitivity to early microstructural changes within the tumor. During the first three chemotherapy cycles—precisely the period with the highest decision-making value—repeated QUS measurements of spectral and scattering parameters (MBF, SS, SI, ESD, EAC), complemented by texture analysis, can reveal early trends in therapeutic response long before they become morphologically apparent in conventional size-based assessments [37,41,97,102,119,122]. In clinical practice, the most efficient approach involves a standardized two-visit protocol: the first examination serves as an early-response screening after the initial treatment cycle, while the second, performed after the third cycle, confirms the response trajectory. This strategy enables rapid identification of non-responders and supports timely adaptation of therapy, reducing the risk of ineffective or unnecessarily prolonged treatment [37,41,119].

Beyond early detection, QUS allows detailed differentiation between complete, partial, and non-response. Parametric and probability maps enable visualization of spatially heterogeneous response patterns, revealing resistant subregions within the tumor. This information has direct therapeutic implications—ranging from early regimen modification or intensification, through targeted rescue biopsies, to optimized surgical planning with more precise margin assessment [97,99,123]. Clinically useful implementations include integrated QUS reports that combine global quantitative metrics with regional response visualization and interpretive commentary addressing discrepancies between ultrasound, MRI, and molecular findings [38,41,100,102].

The clinical potential of adaptive, QUS-guided monitoring is particularly evident in triple-negative breast cancer (TNBC), where rapid tumor kinetics and a narrow therapeutic window necessitate early response evaluation. In HER2-positive disease, QUS can inform treatment de-escalation in patients demonstrating strong early response signatures and conversely support continuation of full-intensity regimens in those with unfavorable early indicators [101,121,124,125]. In luminal subtypes, QUS may aid in adjusting the duration and intensity of NAC, where response dynamics are typically slower and less predictable.

In breast-conserving surgery, QUS provides valuable spatial information on the distribution of residual viable tissue following NAC, facilitating precise resection planning and minimizing the risk of inadequate margins [98,99]. A logical extension of this concept is axillary assessment: serial QUS evaluation of lymph nodes before and during NAC can identify nodal regression or persistence, supporting the choice of sentinel node biopsy over full dissection and guiding targeted axillary dissection (TAD) procedures [126,127]. Furthermore, in metastatic settings, QUS serves as a rapid, repeatable biomarker for monitoring response in ultrasound-accessible lesions (e.g., liver or soft tissue), where early assessment of subsequent treatment lines is clinically valuable [53,100,122].

To transition from research to routine clinical application, three foundational elements must be established. First, standardized protocols for data acquisition and processing—covering fixed presets, transducer selection, calibration procedures, phantom-based normalization, and comprehensive metadata documentation—are required. Second, explicit interpretive frameworks should define quantitative thresholds and composite indices, alongside decision-support algorithms enabling adaptive therapy guidance. Third, robust multicenter validation is essential, ideally through prospective, interventional trials in which treatment adjustments are explicitly informed by QUS findings.

In parallel, the integration of QUS reporting into Picture Archiving and Communication System (PACS) and Hospital Information System (HIS) platforms, structured clinician training, and cost-effectiveness analyses will be crucial. The ultimate value of QUS lies not only in improving predictive precision but also in minimizing unnecessary toxicity, preventing treatment delays, and enabling personalized therapy from the earliest stages of management. Establishing these frameworks is essential for transitioning QUS from an emerging research tool to a clinically actionable instrument in precision oncology.

A more widespread clinical deployment of QUS will depend not only on technical validation but also on its integration into everyday oncology workflows. Key considerations include operator training and competency assessment—particularly in interpreting quantitative pparametric maps rather than conventional B-mode images—together with seamless interoperability between QUS software, PACS, and HISs to enable automated data archiving and reporting. Time efficiency also plays a critical role: whil current analyses are often performed offline, the development of near real-time QUS pipelines could significantly shorten interpretation time and facilitate clinical adoption. From an economic perspective, QUS represents a low-cost, accessible, and repeatable imaging option compared with magnetic resonance imaging (MRI) or contrast-enhanced ultrasound, providing an opportunity to monitor treatment response more frequently without increasing healthcare costs. Importantly, recent oncologic evidence indicates that different patterns of residual disease after neoadjuvant chemotherapy are independently associated with long-term outcomes in breast cancer [128]. By enabling early visualization of microstructural heterogeneity and residual viable tissue, QUS may support prognostic stratification and guide personalized surgical or systemic treatment decisions, thereby enhancing its translational and clinical impact.

### 5.2. Technical Challenges and Standardization Issues

Despite strong clinical evidence supporting the utility of quantitative ultrasound (QUS), several critical challenges must be addressed before this technology can be fully integrated into routine oncological workflows. One of the most significant limitations remains the lack of comprehensive standardization in both data acquisition and signal processing. QUS parameters are inherently sensitive to scanner presets, transducer characteristics, beamforming configurations, and post-processing algorithms, all of which contribute to variability across institutions and ultrasound systems. Although calibration phantoms and harmonization initiatives—such as the Quantitative Imaging Biomarkers Alliance (QIBA) and the Partnership for the Advancement of Quantitative Ultrasound in Medicine (PAQUS)—represent meaningful steps toward consistency, universally accepted clinical protocols have yet to be established.

In recent years, these international initiatives have begun to define reproducibility frameworks for QUS biomarkers. The QIBA Ultrasound Committee has issued consensus profiles specifying acquisition parameters, calibration procedures, and performance metrics for backscatter and attenuation imaging. In parallel, PAQUS promotes benchmarking of inter-system variability through standardized tissue-mimicking phantom studies and encourages the creation of open-access reference datasets. Adoption of such frameworks could substantially reduce variability arising from differences in scanner hardware, transducer design, or post-processing algorithms. To move toward full harmonization, several practical steps are recommended: (1) implementation of cross-platform calibration using phantoms with traceable acoustic properties; (2) establishment of shared repositories of raw RF data enabling reproducibility testing, algorithm benchmarking, and AI training; (3) publication of vendor-neutral analysis pipelines with transparent metadata documentation; and (4) incorporation of standardization protocols in multicenter clinical trials to derive normative QUS reference ranges. Collectively, these actions would enhance the reliability, comparability, and translational readiness of QUS biomarkers across platforms and clinical environments.

Equally important is the interpretation of QUS data, which still lacks validated quantitative thresholds or decision rules for clinical application. Clinicians require standardized indices and structured reporting systems capable of categorizing tumors as “responding” or “non-responding,” rather than providing relative parameter changes without actionable context. Tumor heterogeneity further complicates interpretation, as global mean metrics may obscure spatially localized resistant subregions. Addressing these limitations will require advanced regional analysis tools—such as parametric probability maps and explainable AI algorithms—capable of integrating QUS findings with MRI, pathology, and molecular data in a transparent and interpretable manner suitable for multidisciplinary tumor board discussions.

From a hardware perspective, inter-system variability remains a major translational barrier. Differences in transducer bandwidth, reconstruction filters, dynamic range, and vendor-specific image-formation pipelines may limit cross-platform generalizability. Techniques such as digital phantom calibration, transfer learning, and harmonized data normalization can mitigate these discrepancies, yet robust multicenter validation remains essential to confirm reproducibility. Furthermore, regulatory approval pathways for AI-based QUS applications must align with international Software as a Medical Device (SaMD) standards, ensuring traceability, explain ability, and clinical safety throughout development and implementation.

Another crucial challenge involves the integration of QUS into everyday clinical practice. At present, most analyses are performed offline, outside standard ultrasound workflows, prolonging interpretation time and requiring specialized expertise. Real-time deployment will depend on the development of automated analysis pipelines, validated AI models, and seamless interoperability with Picture Archiving and Communication Systems (PACS) and Hospital Information Systems (HIS). Without such integration, widespread clinical adoption will remain limited. Achieving this transition will require close collaboration among engineers, radiologists, oncologists, and IT specialists to develop user-friendly QUS interfaces and standardized, structured reports aligned with oncologic decision-making pathways.

### 5.3. Organizational and Ethical Challenges

Despite the growing body of supportive evidence, large-scale, prospective, and particularly interventional multicenter trials remain logistically demanding and resource-intensive. Such studies are, however, indispensable for demonstrating that QUS-guided adaptive therapeutic strategies can not only improve patient outcomes but also provide cost-effective benefits in real-world oncology practice. Ethical considerations must likewise be carefully addressed. Modifying or discontinuing systemic therapy based solely on experimental QUS biomarkers carries inherent risks, underscoring the need for transparent validation frameworks, strict patient safety protocols, and ongoing regulatory oversight. Building clinician confidence in QUS-based decision-making will ultimately depend on demonstrating clinical benefit, interpretability, and safety within rigorously controlled multicenter trials.

## 6. Summary and Future Vision

Quantitative ultrasound (QUS) currently stands at the interface between translational imaging research and clinical oncology. Its unique ability to detect early microstructural changes, quantify intratumoral heterogeneity, and integrate multiparametric biomarkers positions it as a promising and biologically meaningful tool for precision medicine. However, to fully realize this potential, further progress requires a coordinated roadmap that addresses both immediate research needs and long-term clinical implementation.

In the near term (short-term), efforts should focus on improving reproducibility, standardization, and interpretability of QUS biomarkers. Establishing harmonized acquisition and processing protocols, validated calibration procedures, and open-access repositories of raw radiofrequency data will be essential for ensuring cross-platform comparability. In parallel, the development of explainable artificial intelligence (XAI) and interpretable radiomic frameworks will enhance transparency and clinical confidence. Prospective reproducibility and repeatability studies across different ultrasound systems should define acceptable variability thresholds and technical benchmarks for clinical reporting, providing the methodological foundation for future validation.

In the longer perspective (long-term), research should advance toward large-scale, multicenter, and interventional clinical trials demonstrating that QUS-guided adaptive therapy can improve oncologic outcomes. These studies should integrate QUS-derived biomarkers into clinical decision-making workflows and evaluate their impact on survival, recurrence, and quality of life. Future directions also encompass health-economic and cost-effectiveness analyses, practical feasibility assessments in diverse clinical settings, and integration of QUS into national cancer registries and reimbursement frameworks. Ultimately, the development of fully automated, real-time QUS systems embedded within PACS and HIS infrastructures will enable broad clinical deployment and support data-driven precision oncology on a population scale.

Only through such coordinated, multidisciplinary efforts—bridging physics, oncology, radiology, and data science—can QUS evolve from an innovative research methodology into a clinically established and widely implemented modality for real-time, adaptive cancer therapy monitoring. Importantly, ongoing trials such as the “Adaptive Neoadjuvant Chemotherapy Based on Quantitative Ultrasound Biomarkers in Locally Advanced Breast Cancer” (ClinicalTrials.gov Identifier: NCT04050228) represent the first crucial step toward this vision, testing whether early QUS-based treatment adaptation can improve therapeutic outcomes and reduce unnecessary toxicity in breast cancer patients.

## Figures and Tables

**Table 1:** Comparative overview of imaging modalities for monitoring response to neoadjuvant chemotherapy (NAC) in breast cancer.

Imaging Modality	Primary Assessment	Sensitivity to Early Biological Changes	Accessibility	Key Limitations
MRI	Morphologic, vascular (contrast-based)	Limited (size, enhancement)	Moderate	Expensive, time-consuming, limited access
CT	Morphological (density)	Limited	High	Ionizing radiation, poor soft-tissue contrast
B-mode	Morphological (echogenicity, size)	Qualitative only	Excellent	Operator-dependent, subjective
Mammography	Morphological (density, calcifications)	None	High	Low accuracy in dense breasts, radiation
Quantitative ultrasound (QUS)	Microstructural (spectral, scattering, attenuation parameters)	Quantitative (RF-derived biomarkers)	Excellent	Lower spatial resolution than MRI; limited standardization

**Table 2:** Quantitative ultrasound (QUS) spectral parameters: technical characteristics, sensitivity to microstructural changes, and clinical relevance.

Parameter	Unit	Sensitivity to Microstructural Changes	Interpretation/Clinical Significance	References
spectral slope (SS)	dB/MHz	High (dominant scatterer size)	Steeper (more negative) slopes imply finer dominant structures; useful for lesion characterization and early therapy monitoring.	[64,65,66,67]
0 MHz intercept (SI)	dB	Moderate-high (backscatter amplitude)	Higher SI indicates stronger impedance fluctuations/overall scatter; sensitive to early treatment-induced cell death (after proper calibration).	[64,65,66,69]
midband fit (MBF)	dB	Moderate-high (backscatter level)	Mean of the fitted log-spectrum within the mid band; increases may reflect apoptosis/early microstructural disorganization; useful for early response.	[50,64,65,66,67]
backscatter coefficient (BSC)	1/(cm·sr)	High (total backscatter intensity)	System-independent physical measure after reference-phantom normalization and attenuation compensation; supports longitudinal, quantitative comparisons.	[49,64,66,69]
effective scatterer diameter (ESD)	mm	High (structure size)	Larger ESD → coarser dominant microstructure (e.g., edema/necrosis); smaller ESD → finer subcellular features (e.g., nuclear condensation). Trends are context-dependent.	[50,64,66]
effective acoustic concentration (EAC)	1/mm^3^	High (number of scatterers)	Higher EAC reflects more or higher-contrast scatterers; may decrease with necrosis or increase with inflammatory/fibrotic changes; interpret with SI/MBF/ESD jointly.	[50,64,66]

**Table 3:** Quantitative ultrasound (QUS) amplitude-based parameters: technical characteristics, sensitivity to microstructural changes, and clinical relevance.

Parameter	Unit	Sensitivity to Microstructural Changes	Interpretation/Clinical Significance	References
Nakagami parameter (m)	-	Moderate–high (homogeneity/clustering)	m ≈ 1: Rayleigh (fully developed speckle, random scattering); m < 1: pre-Rayleigh (sparse/heterogeneous); m > 1: post-Rayleigh (more coherent/ordered microstructure). Useful for tracking organization/fibrosis and malignancy-related heterogeneity.	[48,76,77]
homodyned K distribution	–	High (mixed scatterer populations)	Flexibly models mixtures from Rayleigh-like speckle to signals dominated by strong, discrete scatterers (e.g., fibrous or calcified inclusions coexisting with cellular tissue).	[48,71,72,78]
envelope entropy	bit	High (tissue heterogeneity)	Higher entropy → greater disorder/heterogeneity; decline during NAC may indicate homogenization/acoustic normalization.	[72,79,80]
Kullback–Leibler (KL) divergence	–	Moderate–high (distributional shift vs. reference)	Statistical distance between tumor amplitude histogram and reference tissue (e.g., contralateral/peritumoral); sensitive to subtle microstructural alterations.	[48,74,75]
skewness	–	Moderate (asymmetry/strong scatterers)	Positive skewness suggests presence of strong scatterers (e.g., calcifications); negative skewness indicates predominance of finer structures.	[75]
kurtosis	–	Moderate (tail/peakedness)	Higher kurtosis → more peaked/“uniform” scattering; lower → broader tails/marked variability and heterogeneity.	[75]
effective number of scatterers (ENS)	–	High (effective scatterer population)	Estimates effective scatterer count; decreases with necrosis or structural homogenization during effective NAC.	[81]
generalized gamma distribution parameters (α, β)	–	High (flexible fit to real histograms)	Shape parameters capture changes in microstructural organization; more flexible than Rayleigh/Nakagami for real amplitude histograms.	[78]

**Table 4:** Summary of preclinical QUS applications across experimental models.

Model	QUS Application	Analyzed QUS Parameters	Key Findings	References
Murine tumor models	Monitoring response to chemotherapy, radiotherapy, and targeted therapies; detection of apoptosis and microstructural remodeling	MBF, SS, SI, ESD, EAC	Early detection of microstructural alterations associated with apoptosis and cell death; strong correlation with histopathology	[36,40,58,98,103,104,105]
In vitro cellular models	Assessment of apoptosis and necrosis effects on ultrasonic backscatter	MBF, SS, SI, ESD, EAC	QUS detects morphological changes such as chromatin condensation and nuclear fragmentation during programmed cell death	[47,48,58,103,104]
Acoustic phantoms	Calibration and validation of QUS systems; repeatability and cross-platform testing	BSC, AC, ESD, EAC	Provide standardized reference media for system calibration; enable evaluation of scatterer-related effects on spectral and statistical parameters	[47,48]
Ex vivo tissue models	Validation of QUS parameters in excised tumor specimens	MBF, SS, SI, ESD, EAC	Confirmed correlation between QUS-derived parameters and tissue microstructural organization in resected samples	[47,48,103,104]

MBF—Midband Fit; SS—Spectral Slope; SI—0 Mhz Intercept; ESD—Effective Scatterer Diameter; EAC—Effective Acoustic Concentration; BSC—Backscatter Coefficient.

**Table 5:** Summary of key single-center clinical studies employing quantitative ultrasound (QUS) for monitoring breast cancer response to neoadjuvant chemotherapy.

Year (Journal)	No. of Patients	Assessment Time	Analyzed QUS Parameters	Key Findings	References
2005–2013 (Cancer Res, Clin Cancer Res)	~20–30	Weeks 1–2	MBF, SS, SI	Early rise in QUS parameters among responders; no change in resistant tumors; markers of apoptosis and microstructural remodeling	[58,106]
2018 (PLoS One)	96	Weeks 1, 4, 8	QUS + texture + molecular	Combined QUS, texture, and molecular biomarkers yielded 78–86% accuracy for early response prediction	[41]
2020 (Ultrsound Med Biol)	100100	Weeks 1, 4, 8	QUS + texture (2 scanners)	High repeatability and inter-system consistency in clinical measurements	[108]
2017 (Sci Rep)	56	Pre-NAC baseline	QUS (tumor core + margin)	Baseline QUS predicts NAC response and 5-year recurrence-free survival	[36]
2022 (Cancers)	83	Week 4	QUS + higher-order texture	Early prediction of recurrence; accuracy ≈ 81%	[90]
2019 (PLoS One)	16/24 tumors	After each NAC cycle (1–5)	IBSC + envelope statistics (H-K)	AUC 0.82–0.91 (2nd–3rd cycle); response predicted earlier than size reduction	[109]
2022 (Med Phys)	37 tumors	After 1st and 3rd dose	KLD/KSS from RF envelopes	Non-response detected after 1st dose (AUC ~0.83–0.84); improved accuracy after 3rd (AUC ~0.90–0.91)	[110]
2021 (Cancers)	24	Baseline + 7 days after 1–4 cycles	Quantitative echogenicity	Echogenicity changes correlate with treatment outcome	[74]
2020 (PLoS One)	59	During NAC	QUS radiomics	In-treatment QUS features outperform baseline in predicting pCR	[100]
2022 (Sci Rep)	181	Pre-NAC	Multiparametric QUS with deep learning	Pre-treatment QUS-based deep learning enables accurate response prediction	[46]
2024 (Sci Rep)	174	Pre-NAC	Transfer learning on QUS maps	Transfer learning improves pre-treatment response prediction	[42]
2024 (IEEE Trans Biomed Eng)	56	Adaptive (serial NAC)	QUS-based probability maps	Serial probabilistic mapping enables adaptive response classification	[99]
2025 (Cancers)	100	Weeks 1, 4, 8	QUS + texture model validation	Prospective validation of early prediction model; supports treatment intensification	[111]
Ongoing (ClinicalTrials.gov NCT04050228)	≥100 (planned)	Adaptive NAC	Parametric QUS + radiomics	Ongoing prospective trial of QUS-guided adaptive NAC	[112]

MBF—midband fit; SS—spectral slope; SI—0 Mhz intercept; IBSC—integrated backscatter coefficient; KLD—Kullback–Leibler divergence; KSS—Kolmogorov–Smirnov statistic; NAC—neoadjuvant chemotherapy; pCR—pathologic complete response.

**Table 6:** Integrative QUS-based models predicting response to neoadjuvant chemotherapy (NAC) in breast cancer. The table outlines multimodal and AI-enhanced approaches combining QUS with texture, molecular, clinical, and radiomic data, showing progressive performance gains from early spectral–texture models to recent deep-learning and longitudinal frameworks.

Year (Journal)	No. of Patients/Centers	Assessment Time	Model Type	Key Findings	References
2018 (PLos ONE)	96	Pretreament, Weeks 1, 4, 8, presurgery	QUS + texture + molecular	Combined QUS, texture, and molecular features predicted response with up to 86% accuracy	[41]
2023 (Sci Rep)	208	Pretreatment	QUS, texture derivatives, core-margin, molecular	87% accuracy in predicting NAC response before treatment; supports risk stratification and personalized therapy	[102]
2023 (Academic Radiology)	255	Pretreatment	QUS (B-mode US, SWE), molecular, CNN	LDUR model (B-mode US + SWE): AUC 0.97, Sensitivity 95.5%, Specificity 91.1%	[45]
2023 (Acad Radiol)	112	Pre-, 2nd and 4th NAC cycles	QUS (BUS), SWE, delta radiomics	Model LDUR (BUS + SWE): AUC 0.97, Sens 95.5%, Spec 91.1%	[120]
2024 (Sci Rep)	174100	PretreatmentWeeks 1, 4, 8	QUS parametric maps (core + margin), deep learning	Transfer learning: balanced accuracy 86%, F1-score 0.83; effective prediction of non-responders (NR) vs. responders (RR)	[42]
2025 (J Imaging)	56	Pretreatment	QUS, texture derivatives, molecular subtype	Sensitivity 94%, Specificity 100%	[38]

Abbreviations: QUS—Quantitative Ultrasound; BUS—B-mode Ultrasound; SWE—Shear Wave Elastography; NAC—Neoadjuvant Chemotherapy; Acc—Accuracy; Sens—Sensitivity; Spec—Specificity; AUC—Area Under the Curve; NR—Non-Responder; RR—Responder.

**Table 7:** Summary of multi-institutional studies using QUS to monitor response to neoadjuvant chemotherapy in breast cancer.

Year (Journal)	No. of Patients/Centers	Assessment Time	Analyzed QUS Parameters	Key Findings	References
2018 (PLoS One)	96 patients, multi-institutional	Pretreatment, Weeks 1, 4, 8, pre-surgery	QUS + texture + molecular	Combined QUS, texture, and molecular features predicted response with up to 86% accuracy at week 4	[41]
2020 (Cancer Med)	82 patients, 4 centers	Pretreatment	Pretreatment QUS radiomics	Radiomic features predicted NAC response with 87% accuracy; supported early risk stratification and personalized therapy	[100]
2022 (eClinicalMedicine)	393 patients, 3 hospitals	Pre-treatment, after 1st/2nd NAC cycles	Deep learning on US images	Siamese multi-task network predicted pCR with AUC 0.90–0.99 in external validation	[121]
2024 (Front Oncol)	Randomized multi-center trial100	Pretreatment, Weeks 1, 4	QUS-guided adaptive NAC	QUS-based model prospectively validated; early prediction enabled therapy adaptation	[101]

Abbreviations: QUS—Quantitative Ultrasound; NAC—Neoadjuvant Chemotherapy; AUC—Area Under the Curve; pCR—Pathologic Complete Response; SVM—Support Vector Machine.

## Data Availability

Not applicable.

## References

[1] Harbeck N., Penault-Llorca F., Cortés J., Gnant M., Houssami N., Poortmans P., Ruddy K., Tsang J., Cardoso F. (2019). Breast cancer. Nat. Rev. Dis. Primers.

[2] Wilkinson L., Gathani T. (2021). Understanding breast cancer as a global health concern. Br. J. Radiol..

[3] Bhushan A., Gonsalves A., Menon J.U. (2021). Current state of breast cancer diagnosis, treatment, and theranostics. Pharmaceutics.

[4] Loibl S., Poortmans P., Morrow M., Denkert C., Curigliano G. (2021). Breast cancer. Lancet.

[5] Hong R., Xu B. (2022). Breast cancer: An up-to-date review and future perspectives. Cancer Commun..

[6] Costa B., Amorim I., Gärtner F., Vale N. (2020). Understanding breast cancer: From conventional therapies to repurposed drugs. Eur. J. Pharm. Sci..

[7] Anastasiadi Z., Lianos G., Ignatiadou E., Harissis H., Mitsis M. (2017). Breast cancer in young women: An overview. Updates Surg..

[8] Zhu J., Charkhchi P., Adekunte S., Akbari M. (2023). What is known about breast cancer in young women?. Cancers.

[9] Johnson R., Anders C., Litton J., Ruddy K., Bleyer A. (2018). Breast cancer in adolescents and young adults. Pediatr. Blood Cancer.

[10] Testa U., Castelli G., Pelosi E. (2020). Breast cancer: A molecularly heterogenous disease needing subtype-specific treatments. Med. Sci..

[11] Akram M., Iqbal M., Daniyal M., Khan A.U. (2017). Awareness and current knowledge of breast cancer. Biol. Res..

[12] Carvalho E., Canberk S., Schmitt F., Vale N. (2025). Molecular subtypes and mechanisms of breast cancer: Precision medicine approaches for targeted therapies. Cancers.

[13] Yin L., Duan J.J., Bian X.W., Yu S.C. (2020). Triple-negative breast cancer molecular subtyping and treatment progress. Breast Cancer Res..

[14] Prat A., Pineda E., Adamo B., Galván P., Fernández A., Gaba L., Diez M., Viladdot M., Arance A., Muñoz M. (2015). Clinical implications of the intrinsic molecular subtypes of breast cancer. Breast.

[15] Russnes H.G., Lingjærde O.C., Børresen-Dale A.L., Caldas C. (2017). Breast cancer molecular stratification: From intrinsic subtypes to integrative clusters. Am. J. Pathol..

[16] Lehmann B.D., Jovanović B., Chen X., Estrada M.V., Johnson K.N., Shyr Y., Moses H.L., Sanders M.E., Pietenpol J.A. (2016). Refinement of triple-negative breast cancer molecular subtypes: Implications for neoadjuvant chemotherapy selection. PLoS ONE.

[17] Bianchini G., Balko J.M., Mayer I.A., Sanders M.E., Gianni L. (2016). Triple-negative breast cancer: Challenges and opportunities of a heterogeneous disease. Nat. Rev. Clin. Oncol..

[18] Garrido-Castro A.C., Lin N.U., Polyak K. (2019). Insights into molecular classifications of triple-negative breast cancer: Improving patient selection for treatment. Cancer Discov..

[19] Krijgsman O., Roepman P., Zwart W., Helleman J., Berns E.M., van’t Veer L.J. (2012). A diagnostic gene profile for molecular subtyping of breast cancer associated with treatment response. Breast Cancer Res. Treat..

[20] Wolf D.M., Yau C., Wulfkuhle J., Brown-Swigart L., Gallagher R.I., Lee P.R.E., Zhu Z., Magbanua M.J., Sayaman R., O’Grady N. (2022). Redefining breast cancer subtypes to guide treatment prioritization and maximize response: Predictive biomarkers across 10 cancer therapies. Cancer Cell.

[21] Łukasiewicz S., Czeczelewski M., Forma A., Baj J., Sitarz R., Stanisławek A. (2021). Breast cancer—Epidemiology, risk factors, classification, prognostic markers, and current treatment strategies—An updated review. Cancers.

[22] Vicini E., Galimberti V., Leonardi M.C., Kahler-Ribeiro-Fontana S., Polizzi A., Petitto S., Pagan E., Bagnardi V., Montagna E., Cavallone M. (2025). Shifting from axillary dissection to targeted axillary surgery after neoadjuvant treatment: The evolving management of occult breast cancer in a monoinstitutional series of 114 patients. Breast Cancer Res. Treat..

[23] Cipolla C., Gebbia V., D’Agati E., Greco M., Mesi C., Scandurra G., Valerio M.R. (2024). Comprehensive axillary management of clinically node-positive (cN+) breast cancer patients: A narrative review on neoadjuvant chemotherapy. Cancers.

[24] Derks M., van de Velde C.J.H. (2018). Neoadjuvant chemotherapy in breast cancer: More than just downsizing. Lancet Oncol..

[25] Franceschini G., Di Leone A., Natale M., Sanchez M.A., Masetti R. (2018). Conservative surgery after neoadjuvant chemotherapy in patients with operable breast cancer. Ann. Ital. Chir..

[26] King T.A., Morrow M. (2015). Surgical issues in patients with breast cancer receiving neoadjuvant chemotherapy. Nat. Rev. Clin. Oncol..

[27] Fowler A.M., Mankoff D.A., Joe B.N. (2017). Imaging neoadjuvant therapy response in breast cancer. Radiology.

[28] Romeo V., Accardo G., Perillo T., Basso L., Garbino N., Nicolai E., Maurea S., Salvatore M. (2021). Assessment and prediction of response to neoadjuvant chemotherapy in breast cancer: A comparison of imaging modalities and future perspectives. Cancers.

[29] Portnow L.H., Kochkodan-Self J.M., Maduram A., Barrios M., Onken A.M., Hong X., Mittendorf E.A., Giess C.S., Chikarmane S.A. (2023). Multimodality imaging review of HER2-positive breast cancer and response to neoadjuvant chemotherapy. Radiographics.

[30] Rezai M., Kraemer S. (2017). Breast-conserving surgery after neoadjuvant therapy. Breast Cancer: Innovations in Research and Management.

[31] Schott A.F., Hayes D.F. (2012). Defining the benefits of neoadjuvant chemotherapy for breast cancer. J. Clin. Oncol..

[32] Eisenhauer E.A., Therasse P., Bogaerts J., Schwartz L.H., Sargent D., Ford R., Dancey J., Arbuck S., Gwyther S., Mooney M. (2009). New response evaluation criteria in solid tumours: Revised RECIST guideline (version 1.1). Eur. J. Cancer.

[33] Lobbes M.B.I., Prevos R., Smidt M.L., Tjan-Heijnen V.C.G., van Goethem M., Schipper R.J., Beets-Tan R.G.H., Wildberger J.E. (2013). The role of magnetic resonance imaging in assessing residual disease and pathologic complete response in breast cancer patients receiving neoadjuvant chemotherapy: A systematic review. Eur. J. Radiol..

[34] Shi Z., Chu C., Liu Y., Zhang L., Zhu L., Liu Y., Liang C., Lu C., Cui Y., Han C. (2023). MRI-based quantification of intratumoral heterogeneity for predicting treatment response to neoadjuvant chemotherapy in breast cancer. Radiology.

[35] Yeh E., Slanetz P., Kopans D.B., Rafferty E., Georgian-Smith D., Moore R.H., Kuter I., Taghian A. (2005). Prospective comparison of mammography, sonography, and MRI in patients undergoing neoadjuvant chemotherapy for palpable breast cancer. AJR Am. J. Roentgenol..

[36] Tadayyon H., Sannachi L., Gangeh M.J., Kim C., Ghandi S., Trudeau M., Pritchard K., Tran W.T., Slodkowska E., Sadeghi-Naini A. (2017). A priori prediction of neoadjuvant chemotherapy response and survival in breast cancer patients using quantitative ultrasound. Sci. Rep..

[37] Klimonda Z., Karwat P., Dobruch-Sobczak K., Piotrzkowska-Wróblewska H., Litniewski J. On the assessment of local tumor response to neoadjuvant chemotherapy. Proceedings of the 2023 IEEE International Ultrasonics Symposium (IUS).

[38] Chan A.W., Sannachi L., Moore-Palhares D., Dasgupta A., Gandhi S., Pezo R., Eisen A., Warner E., Wright F.C., Look Hong N. (2025). Validation of quantitative ultrasound and texture derivative analyses-based model for upfront prediction of neoadjuvant chemotherapy response in breast cancer. J. Imaging.

[39] Piotrzkowska-Wróblewska H., Dobruch-Sobczak K., Gumowska M., Litniewski J. Changes in quantitative ultrasound imaging as the predictor of response to neoadjuvant chemotherapy in patients with breast cancer. Proceedings of the IEEE International Ultrasonics Symposium (IUS).

[40] Sadeghi-Naini A., Sannachi L., Tadayyon H., Tran W., Slodkowska E., Trudeau M., Gandhi S., Pritchard K., Kolios M.C., Czarnota G. (2017). Chemotherapy-response monitoring of breast cancer patients using quantitative ultrasound-based intra-tumour heterogeneities. Sci. Rep..

[41] Sannachi L., Gangeh M., Tadayyon H., Sadeghi-Naini A., Gandhi S., Wright F., Slodkowska E., Curpen B., Tran W., Czarnota G. (2018). Response monitoring of breast cancer patients receiving neoadjuvant chemotherapy using quantitative ultrasound, texture, and molecular features. PLoS ONE.

[42] Falou O., Sannachi L., Haque M., Czarnota G., Kolios M.C. (2024). Transfer learning of pre-treatment quantitative ultrasound multi-parametric images for the prediction of breast cancer response to neoadjuvant chemotherapy. Sci. Rep..

[43] Yu F., Miao S., Li C., Hang J., Deng J., Ye X., Liu Y. (2023). Pretreatment ultrasound-based deep learning radiomics model for the early prediction of pathologic response to neoadjuvant chemotherapy in breast cancer. Eur. Radiol..

[44] Gu J., Tong T., He C., Xu M., Yang X., Tian J., Jiang T., Wang K. (2021). Deep learning radiomics of ultrasonography can predict response to neoadjuvant chemotherapy in breast cancer at an early stage of treatment: A prospective study. Eur. Radiol..

[45] Huang J.X., Shi J., Ding S., Zhang H.L., Wang X.Y., Lin S.Y., Xu Y.F., Wei M.J., Liu L.Z., Pei X. (2023). Deep learning model based on dual-modal ultrasound and molecular data for predicting response to neoadjuvant chemotherapy in breast cancer. Acad. Radiol..

[46] Taleghamar H., Jalalifar S., Czarnota G., Sadeghi-Naini A. (2022). Deep learning of quantitative ultrasound multi-parametric images at pre-treatment to predict breast cancer response to chemotherapy. Sci. Rep..

[47] Oelze M.L., Mamou J. (2016). Review of quantitative ultrasound: Envelope statistics and backscatter coefficient imaging and contributions to diagnostic ultrasound. IEEE Trans. Ultrason. Ferroelectr. Freq. Control.

[48] Cloutier G., Destrempes F., Yu F., Tang A. (2021). Quantitative ultrasound imaging of soft biological tissues: A primer for radiologists and medical physicists. Insights Imaging.

[49] Destrempes F., Franceschini E., Yu F., Cloutier G. (2016). Unifying concepts of statistical and spectral quantitative ultrasound techniques. IEEE Trans. Med. Imaging.

[50] Lavarello R., Oelze M. (2011). Quantitative ultrasound estimates from populations of scatterers with continuous size distributions. IEEE Trans. Ultrason. Ferroelectr. Freq. Control..

[51] Sadeghi-Naini A., Suraweera H., Tran W., Hadizad F., Bruni G., Rastegar R., Curpen B., Czarnota G.J. (2017). Breast-lesion characterization using textural features of quantitative ultrasound parametric maps. Sci. Rep..

[52] Klimonda Z., Karwat P., Dobruch-Sobczak K., Piotrzkowska-Wróblewska H., Litniewski J. (2019). Breast-lesions characterization using quantitative ultrasound features of peritumoral tissue. Sci. Rep..

[53] Sharma D., Osapoetra L., Czarnota G. (2022). Implementation of non-invasive quantitative ultrasound in clinical cancer imaging. Cancers.

[54] Nizam N., Ara S., Hasan M.K. (2019). Classification of breast lesions using quantitative ultrasound biomarkers. Biomed. Signal Process. Control.

[55] Jia F., Sun S., Li J., Wang W., Huang H., Hu X., Pan S., Chen W., Shen L., Yao Y. (2024). Neoadjuvant chemotherapy-induced remodeling of human hormonal receptor-positive breast cancer revealed by single-cell RNA sequencing. Cancer Lett..

[56] Hoshina H., Sakatani T., Kawamoto Y., Ohashi R., Takei H. (2024). Cytomorphological disparities in invasive breast cancer cells following neoadjuvant endocrine therapy and chemotherapy. Pathobiology.

[57] Derouane F., Ambroise J., Van Marcke C., Van Bockstal M., Berlière M., Galant C., Dano H., Lougué M., Benidovskaya E., Jerusalem G. (2025). Response to neoadjuvant chemotherapy in early breast cancers is associated with epithelial-mesenchymal transition and tumor-infiltrating lymphocytes. Mol. Oncol..

[58] Sadeghi-Naini A., Papanicolau N., Falou O., Zubovits J., Dent R., Verma S., Trudeau M., Boileau J., Spayne J., Iradji S. (2013). Quantitative ultrasound evaluation of tumor cell death response in locally advanced breast cancer patients receiving chemotherapy. Clin. Cancer Res..

[59] Tadayyon H., Sadeghi-Naini A., Wirtzfeld L., Wright F.C., Czarnota G. (2014). Quantitative ultrasound characterization of locally advanced breast cancer by estimation of its scatterer properties. Med. Phys..

[60] Sannachi L., Gangeh M., Tadayyon H., Gandhi S., Wright F.C., Slodkowska E., Curpen B., Sadeghi-Naini A., Tran W., Czarnota G.J. (2019). Breast cancer treatment response monitoring using quantitative ultrasound and texture analysis: Comparative analysis of analytical models. Transl. Oncol..

[61] Sannachi L., Tadayyon H., Sadeghi-Naini A., Tran W.T., Gandhi S., Wright F.C., Oelze M., Czarnota G.J. (2015). Non-invasive evaluation of breast cancer response to chemotherapy using quantitative ultrasonic backscatter parameters. Med. Image Anal..

[62] Wang H., Zhao C., Santa-Maria C., Emens L., Popel A. (2022). Dynamics of tumor-associated macrophages in a quantitative systems pharmacology model of immunotherapy in triple-negative breast cancer. iScience.

[63] Du Terrail O., Leopold A., Joly C., Béguier C., Andreux M., Maussion C., Schmauch B., Tramel E., Bendjebbar E., Zaslavskiy M. (2023). Federated learning for predicting histological response to neoadjuvant chemotherapy in triple-negative breast cancer. Nat. Med..

[64] Dasgupta S., Feleppa E.J., Mamou J., Rondeau M. (2006). Validating the theoretical framework relating ultrasonic spectrum-analysis parameters to scatterer properties. J. Acoust. Soc. Am..

[65] Mamou J., Feleppa E.J., Dasgupta S., Rondeau M.J. 2G-3 Validating the Theory Relating Ultrasonic Spectral-Parameter Values to Scatterer Properties. Proceedings of the 2006 IEEE Ultrasonics Symposium.

[66] Lizzi F.L., Astor M., Liu T., Deng C., Coleman D.J., Silverman R.H. (1997). Ultrasonic spectrum analysis for tissue assays and therapy evaluation. Int. J. Imaging Syst. Technol..

[67] Trumpaitis J., Jurkonis R., Imbrasiene D., Grizickaitė A., Paunksnis A. (2014). Application of ultrasound spectral analysis for intraocular tissues differentiation. J. Vibroeng..

[68] Dasgupta S., Feleppa E.J. 4C-1 Empirical validation of the theoretical frameworks underlying ultrasound scattering in tissue. Proceedings of the 2007 IEEE Ultrasonics Symposium Proceedings.

[69] Destrempes F., Cloutier G. (2023). Review of envelope statistics models for quantitative ultrasound imaging and tissue characterization. Adv. Exp. Med. Biol..

[70] Oelze M.L., Zachary J.F. (2006). Examination of cancer in mouse models using high-frequency quantitative ultrasound. Ultrasound Med. Biol..

[71] Tehrani A.K.Z., Cloutier G., Tang A., Rosado-Méndez I., Rivaz H. (2024). Homodyned K-distribution parameter estimation in quantitative ultrasound: Autoencoder and Bayesian neural network approaches. IEEE Trans. Ultrason. Ferroelectr. Freq. Control.

[72] Mori S., Arakawa M., Kanai H., Hachiya H. (2023). Quantification of limitations in statistical analysis of ultrasound echo envelope amplitudes. Jpn. J. Appl. Phys..

[73] Omura M., Yoshida K., Kohta M., Kubo T., Ishiguro T., Kobayashi K., Hozumi N., Yamaguchi T. (2016). Tissue characterization of skin ulcer for bacterial infection by multiple statistical analysis of echo amplitude envelope. Jpn. J. Appl. Phys..

[74] Dobruch-Sobczak K., Piotrzkowska-Wróblewska H., Karwat P., Klimonda Z., Markiewicz-Grodzicka E., Litniewski J. (2021). Quantitative assessment of the echogenicity of a breast tumor predicts the response to neoadjuvant chemotherapy. Cancers.

[75] Nasief H., Rosado-Méndez I., Zagzebski J., Hall T. (2019). A quantitative ultrasound-based multi-parameter classifier for breast masses. Ultrasound Med. Biol..

[76] Tsui P.H., Yeh C.K., Liao Y.Y., Chang C.C., Kuo W.H., Chang K.J., Chen C.N. (2010). Ultrasonic Nakagami imaging: A strategy to visualize the scatterer properties of benign and malignant breast tumors. Ultrasound Med. Biol..

[77] Shankar P.M., Dumane V.A., Reid J.M., Genis V., Forsberg F., Piccoli C.W., Goldberg B.B. (2001). Classification of ultrasonic B-mode images of breast masses using Nakagami distribution. IEEE Trans. Ultrason. Ferroelectr. Freq. Control.

[78] Destrempes F., Cloutier G. (2010). A critical review and uniformized representation of statistical distributions modeling the ultrasound echo envelope. Ultrasound Med. Biol..

[79] Tsui P.H., Chen C.K., Kuo W.H., Chang K.J., Fang J., Ma H.Y., Chou D. (2017). Small-window parametric imaging based on information entropy for ultrasound tissue characterization. Sci. Rep..

[80] Tsui P.H. (2015). Ultrasound detection of scatterer concentration by weighted entropy. Entropy.

[81] Vajihi Z., Rosado-Méndez I., Hall T., Rivaz H. (2018). Low variance estimation of backscatter quantitative ultrasound parameters using dynamic programming. IEEE Trans. Ultrason. Ferroelectr. Freq. Control.

[82] Wang J., Gu Y., Zhan Y., Li R., Bi Y., Gao L., Wu X., Shao J., Chen Y., Ye L. (2025). Intratumoral and peritumoral ultrasound radiomics analysis for predicting HER2-low expression in HER2-negative breast cancer patients: A retrospective analysis of dual-central study. Discov. Oncol..

[83] Nam K., Zagzebski J.A., Hall T.J. (2013). Quantitative assessment of in vivo breast masses using ultrasound attenuation and backscatter. Ultrason. Imaging.

[84] D’Astous F., Foster F.S. (1986). Frequency dependence of ultrasound attenuation and backscatter in breast tissue. Ultrasound Med. Biol..

[85] Huang S.W., Li P.C. (2005). Ultrasonic computed tomography reconstruction of the attenuation coefficient using a linear array. IEEE Trans. Ultrason. Ferroelectr. Freq. Control.

[86] Sarno D., Baker C., Curtis S., Hodnett M., Zeqiri B. (2022). In vivo measurements of the bulk ultrasonic attenuation coefficient of breast tissue using a novel phase-insensitive receiver. IEEE Trans. Ultrason. Ferroelectr. Freq. Control.

[87] Dobruch-Sobczak K., Piotrzkowska-Wróblewska H., Klimonda Z., Karwat P., Roszkowska-Purska K., Clauser P., Baltzer P., Litniewski J. (2021). Multiparametric ultrasound examination for response assessment in breast cancer patients undergoing neoadjuvant therapy. Sci. Rep..

[88] Tadayyon H., Sannachi L., Czarnota G.J. (2014). Quantitative ultrasound monitoring of breast tumor response to chemotherapy by analysis of frequency-dependent attenuation and backscattered power. SPIE Med. Imaging.

[89] Oh S., Kim M.G., Kim Y., Jung G.J., Kwon H., Bae H.M. Spatio-temporal quantitative ultrasound imaging for breast cancer identification. Proceedings of the 2023 IEEE 20th International Symposium on Biomedical Imaging (ISBI).

[90] Bhardwaj D., Dasgupta A., Dicenzo D., Brade S., Fatima K., Quiaoit K., Trudeau M., Gandhi S., Eisen A., Wright F. (2022). Early changes in quantitative ultrasound imaging parameters during neoadjuvant chemotherapy to predict recurrence in patients with locally advanced breast cancer. Cancers.

[91] Alberico D., Sannachi L., Anzola Pena M.L., Yip J., Osapoetra L.O., Halstead S., DiCenzo D., Gandhi S., Wright F., Oelze M. (2025). Quantitative ultrasound texture analysis of breast tumors: A comparison of a cart-based and a wireless ultrasound scanner. J. Imaging.

[92] Osapoetra L., Sannachi L., Dicenzo D., Quiaoit K., Fatima K., Czarnota G. (2020). Breast lesion characterization using quantitative ultrasound (QUS) and derivative texture methods. Transl. Oncol..

[93] Alvarenga A., Pereira W., Infantosi A., Azevedo C. (2007). Complexity curve and grey level co-occurrence matrix in the texture evaluation of breast tumor on ultrasound images. Med. Phys..

[94] Gómez W., Pereira W., Infantosi A. (2012). Analysis of co-occurrence texture statistics as a function of gray-level quantization for classifying breast ultrasound. IEEE Trans. Med. Imaging.

[95] Djunaidi K., Agtriadi H.B., Kuswardani D., Purwanto Y. (2021). Gray level co-occurrence matrix feature extraction and histogram in breast cancer classification with ultrasonographic imagery. Indones. J. Electr. Eng. Comput. Sci..

[96] Czarnota G.J., Tadayyon H., Gangeh M.J., Sannachi L., Sadeghi-Naini A., Tran W.T. Quantitative ultrasound and texture predictors of breast tumour response to chemotherapy. Proceedings of the 2018 IEEE International Ultrasonics Symposium (IUS).

[97] Dasgupta A., Brade S., Sannachi L., Quiaoit K., Fatima K., Dicenzo D., Osapoetra L., Saifuddin M., Trudeau M., Gandhi S. (2020). Quantitative ultrasound radiomics using texture derivatives in prediction of treatment response to neo-adjuvant chemotherapy for locally advanced breast cancer. Oncotarget.

[98] Taleghamar H., Moghadas-Dastjerdi H., Czarnota G., Sadeghi-Naini A. (2021). Characterizing intra-tumor regions on quantitative ultrasound parametric images to predict breast cancer response to chemotherapy at pre-treatment. Sci. Rep..

[99] Karwat P., Piotrzkowska-Wróblewska H., Klimonda Z., Dobruch-Sobczak K., Litniewski J. (2024). Monitoring breast cancer response to neoadjuvant chemotherapy using probability maps derived from quantitative ultrasound parametric images. IEEE Trans. Biomed. Eng..

[100] Dicenzo D., Quiaoit K., Fatima K., Bhardwaj D., Sannachi L., Gangeh M., Sadeghi-Naini A., Dasgupta A., Kolios M.C., Trudeau M. (2020). Quantitative ultrasound radiomics in predicting response to neoadjuvant chemotherapy in patients with locally advanced breast cancer: Results from multi-institutional study. Cancer Med..

[101] Dasgupta A., Dicenzo D., Sannachi L., Gandhi S., Pezo R.C., Eisen A., Tran W.T., Look Hong N., Wright F.C., Curpen B. (2024). Quantitative ultrasound radiomics guided adaptive neoadjuvant chemotherapy in breast cancer: Early results from a randomized feasibility study. Front. Oncol..

[102] Sannachi L., Osapoetra L., Dicenzo D., Halstead S., Wright F., Look-Hong N., Slodkowska E., Gandhi S., Curpen B., Kolios M.C. (2023). A priori prediction of breast cancer response to neoadjuvant chemotherapy using quantitative ultrasound, texture derivative and molecular subtype. Sci. Rep..

[103] Czarnota G.J., Kolios M.C., Abraham J., Portnoy M., Ottensmeyer F.P., Hunt J.W., Sherar M.D. (1999). Ultrasound imaging of apoptosis: High-resolution non-invasive monitoring of programmed cell death in vitro, in situ and in vivo. Br. J. Cancer.

[104] Sharma D., Carter H., Sannachi L., Cui W., Giles A., Saifuddin M., Czarnota G.J. (2023). Quantitative ultrasound for evaluation of tumour response to ultrasound-microbubbles and hyperthermia. Technol. Cancer Res. Treat..

[105] Czarnota G.J. (2005). Role of ultrasound in the detection of apoptosis. Eur. J. Nucl. Med. Mol. Imaging.

[106] Kolios M.C., Czarnota G.J., Lee M., Hunt J.W., Sherar M.D. (2002). Ultrasonic spectral parameter characterization of apoptosis. Ultrasound Med. Biol..

[107] Dighe S., Shinde R., Shinde S., Verma P. (2022). Assessment of response of neoadjuvant chemotherapy in carcinoma breast patients by high-frequency ultrasound. J. Fam. Med. Prim. Care.

[108] Sannachi L., Gangeh M., Naini A.S., Bhargava P., Jain A., Tran W.T., Czarnota G.J. (2020). Quantitative ultrasound monitoring of breast tumour response to neoadjuvant chemotherapy: Comparison of results among clinical scanners. Ultrasound Med. Biol..

[109] Piotrzkowska-Wróblewska H., Dobruch-Sobczak K., Klimonda Z., Karwat P., Roszkowska-Purska K., Gumowska M., Litniewski J. (2019). Monitoring breast cancer response to neoadjuvant chemotherapy with ultrasound signal statistics and integrated backscatter. PLoS ONE.

[110] Klimonda Z., Karwat P., Dobruch-Sobczak K., Piotrzkowska-Wróblewska H., Litniewski J. (2022). Assessment of breast cancer response to neoadjuvant chemotherapy based on ultrasound backscattering envelope statistics. Med. Phys..

[111] Moore-Palhares D., Sannachi L., Chan A.W., Dasgupta A., DiCenzo D., Gandhi S., Pezo R., Eisen A., Warner E., Wright F. (2025). Validation of a quantitative ultrasound texture analysis model for early prediction of neoadjuvant chemotherapy response in breast cancer: A prospective serial imaging study. Cancers.

[112] Use of Quantitative Ultrasound to Guide Adaptive Chemotherapy Among Women with Breast Cancer. ClinicalTrials.gov Identifier:NCT04050228. NCT04050228.

[113] Wenwen J., Jiang Z., Liu J., Liu D., Li Y., He Y., Zhao H., Li L., Zhu Y., Long Q. (2025). Integrating ultrasound radiomics and clinicopathological features for machine learning-based survival prediction in patients with nonmetastatic triple-negative breast cancer. BMC Cancer.

[114] Mao N., Dai Y., Zhou H., Lin F., Zheng T., Li Z., Yang P., Zhao F., Li Q., Wang W. (2025). A multimodal and fully automated system for prediction of pathological complete response to neoadjuvant chemotherapy in breast cancer. Sci. Adv..

[115] Yang M., Liu H., Dai Q., Yao L., Zhang S., Wang Z., Li J., Duan Q. (2022). Treatment response prediction using ultrasound-based pre-, post-early, and delta radiomics in neoadjuvant chemotherapy in breast cancer. Front. Oncol..

[116] Byra M., Dobruch-Sobczak K., Klimonda Z., Piotrzkowska-Wróblewska H., Litniewski J. (2021). Early prediction of response to neoadjuvant chemotherapy in breast cancer sonography using Siamese convolutional neural networks. IEEE J. Biomed. Health Inform..

[117] Byra M., Dobruch-Sobczak K., Piotrzkowska-Wróblewska H., Klimonda Z., Litniewski J. (2022). Prediction of response to neoadjuvant chemotherapy in breast cancer with recurrent neural networks and raw ultrasound signals. Phys. Med. Biol..

[118] Feng X., Shi Y., Wu M., Cui G., Du Y., Yang J., Xu Y., Wang W., Liu F. (2025). Predicting the efficacy of neoadjuvant chemotherapy in breast cancer patients based on ultrasound longitudinal temporal-depth network fusion model. Breast Cancer Res..

[119] Huang J.X., Wu L., Wang X.Y., Lin S.Y., Xu Y.F., Wei M.J., Pei X.Q. (2024). Delta radiomics based on longitudinal dual-modal ultrasound can early predict response to neoadjuvant chemotherapy in breast cancer patients. Acad. Radiol..

[120] Gu J., Zhong X., Fang C., Lou W., Fu P., Woodruff H., Wang B., Jiang T., Lambin P. (2023). Deep learning of multimodal ultrasound: Stratifying the response to neoadjuvant chemotherapy in breast cancer before treatment. Oncologist.

[121] Liu Y., Wang Y., Wang Y., Xie Y., Cui Y., Feng S., Yao M., Qiu B., Shen W., Chen D. (2022). Early prediction of treatment response to neoadjuvant chemotherapy based on longitudinal ultrasound images of HER2-positive breast cancer patients by Siamese multi-task network: A multicentre, retrospective cohort study. eClinicalMedicine.

[122] Sadeghi-Naini A., Sannachi L., Pritchard K., Trudeau M., Gandhi S., Wright F., Zubovits J., Yaffe M., Kolios M., Czarnota G. (2014). Early prediction of therapy responses and outcomes in breast cancer patients using quantitative ultrasound spectral texture. Oncotarget.

[123] Tadayyon H., Sannachi L., Gangeh M., Sadeghi-Naini A., Tran W., Trudeau M., Pritchard K., Ghandi S., Verma S., Czarnota G. (2016). Quantitative ultrasound assessment of breast tumor response to chemotherapy using a multi-parameter approach. Oncotarget.

[124] Adrada B.E., Candelaria R., Moulder S., Thompson A., Wei P., Whitman G., Valero V., Litton J.K., Santiago L., Scoggins M.E. (2021). Early ultrasound evaluation identifies excellent responders to neoadjuvant systemic therapy among patients with triple-negative breast cancer. Cancer.

[125] Peréz-García J., Gebhart G., Ruíz Borrego M., Stradella A., Bermejo B., Schmid P., Marmé F., Escrivá-de-Romani S., Calvo L., Ribelles N. (2021). Chemotherapy de-escalation using an 18F-FDG-PET-based pathological response-adapted strategy in patients with HER2-positive early breast cancer (PHERGain): A multicentre, randomised, open-label, non-comparative, phase 2 trial. Lancet Oncol..

[126] Fu Y., Lei Y.T., Huang Y., Mei F., Wang S., Yan K., Wang Y.H., Ma Y.H., Cui L.G. (2024). Longitudinal ultrasound-based AI model predicts axillary lymph node response to neoadjuvant chemotherapy in breast cancer: A multicenter study. Eur. Radiol..

[127] Li Z., Tong Y., Chen X., Shen K. (2022). Accuracy of ultrasonographic changes during neoadjuvant chemotherapy to predict axillary lymph node response in clinical node-positive breast cancer patients. Front. Oncol..

[128] Tinterri C., Fernandes B., Zambelli A., Sagona A., Barbieri E., Di Maria Grimaldi S., Darwish S.S., Jacobs F., De Carlo C., Iuzzolino M. (2024). The Impact of Different Patterns of Residual Disease on Long-Term Oncological Outcomes in Breast Cancer Patients Treated with Neo-Adjuvant Chemotherapy. Cancers.

