# Correction: Piotrzkowska-Wróblewska, H. The Role of Quantitative Ultrasound in Monitoring Neoadjuvant Chemotherapy in Breast Cancer: A Narrative Review. *Cancers* 2025, *17*, 3676

**DOI:** 10.3390/cancers18142338

**Published:** 2026-07-20

**Authors:** Hanna Piotrzkowska-Wróblewska

**Affiliations:** Ultrasound Department, Institute of Fundamental Technological Research, Polish Academy of Sciences, 02-106 Warsaw, Poland; hpiotrzk@ippt.pan.pl

**Error in References**

In the original publication [1], there were mistakes in several references as published. The errors concerned typographical mistakes in author names, omitted co-authors, inconsistencies in reference formatting, and incorrect article titles in selected references. The corrected references appear below.

12. Carvalho, E.; Canberk, S.; Schmitt, F.; Vale, N. Molecular subtypes and mechanisms of breast cancer: Precision medicine approaches for targeted therapies. *Cancers* **2025**, *17*, 1102. https://doi.org/10.3390/cancers17071102.
20. Wolf, D.M.; Yau, C.; Wulfkuhle, J.; Brown-Swigart, L.; Gallagher, R.I.; Lee, P.R.E.; Zhu, Z.; Magbanua, M.J.; Sayaman, R.; O’Grady, N.; et al. Redefining breast cancer subtypes to guide treatment prioritization and maximize response: Predictive biomarkers across 10 cancer therapies. *Cancer Cell* **2022**, *40*, 609–623.e6. https://doi.org/10.1016/j.ccell.2022.05.005.
22. Vicini, E.; Galimberti, V.; Leonardi, M.C.; Kahler-Ribeiro-Fontana, S.; Polizzi, A.; Petitto, S.; Pagan, E.; Bagnardi, V.; Montagna, E.; Cavallone, M.; et al. Shifting from axillary dissection to targeted axillary surgery after neoadjuvant treatment: The evolving management of occult breast cancer in a monoinstitutional series of 114 patients. *Breast Cancer Res. Treat.* **2025**, *210*, 661–672. https://doi.org/10.1007/s10549-024-07604-3.
31. Schott, A.F.; Hayes, D.F. Defining the benefits of neoadjuvant chemotherapy for breast cancer. *J. Clin. Oncol.* **2012**, *30*, 1747–1749. https://doi.org/10.1200/JCO.2011.41.3161.
36. Tadayyon, H.; Sannachi, L.; Gangeh, M.J.; Kim, C.; Ghandi, S.; Trudeau, M.; Pritchard, K.; Tran, W.T.; Slodkowska, E.; Sadeghi-Naini, A.; et al. A priori prediction of neoadjuvant chemotherapy response and survival in breast cancer patients using quantitative ultrasound. *Sci. Rep.* **2017**, *7*, 45733. https://doi.org/10.1038/srep45733.
60. Sannachi, L.; Gangeh, M.; Tadayyon, H.; Gandhi, S.; Wright, F.C.; Slodkowska, E.; Curpen, B.; Sadeghi-Naini, A.; Tran, W.; Czarnota, G.J. Breast cancer treatment response monitoring using quantitative ultrasound and texture analysis: Comparative analysis of analytical models. *Transl. Oncol.* **2019**, *12*, 1271–1281. https://doi.org/10.1016/j.tranon.2019.06.004.
65. Mamou, J.; Feleppa, E.J.; Dasgupta, S.; Rondeau, M.J. 2G-3 Validating the Theory Relating Ultrasonic Spectral-Parameter Values to Scatterer Properties. In Proceedings of the 2006 IEEE Ultrasonics Symposium, Vancouver, BC, Canada, 2–6 October 2006; pp. 633–636. https://doi.org/10.1109/ULTSYM.2006.173.
76. Tsui, P.H.; Yeh, C.K.; Liao, Y.Y.; Chang, C.C.; Kuo, W.H.; Chang, K.J.; Chen, C.N. Ultrasonic Nakagami imaging: A strategy to visualize the scatterer properties of benign and malignant breast tumors. *Ultrasound Med. Biol.* **2010**, *36*, 209–217. https://doi.org/10.1016/j.ultrasmedbio.2009.10.006.
77. Shankar, P.M.; Dumane, V.A.; Reid, J.M.; Genis, V.; Forsberg, F.; Piccoli, C.W.; Goldberg, B.B. Classification of ultrasonic B-mode images of breast masses using Nakagami distribution. *IEEE Trans. Ultrason. Ferroelectr. Freq. Control* **2001**, *48*, 569–580. https://doi.org/10.1109/58.911740.
79. Tsui, P.H.; Chen, C.K.; Kuo, W.H.; Chang, K.J.; Fang, J.; Ma, H.Y.; Chou, D. Small-window parametric imaging based on information entropy for ultrasound tissue characterization. *Sci. Rep.* **2017**, *7*, 41004. https://doi.org/10.1038/srep41004.
101. Dasgupta, A.; Dicenzo, D.; Sannachi, L.; Gandhi, S.; Pezo, R.C.; Eisen, A.; Tran, W.T.; Look Hong, N.; Wright, F.C.; Curpen, B.; et al. Quantitative ultrasound radiomics guided adaptive neoadjuvant chemotherapy in breast cancer: Early results from a randomized feasibility study. *Front. Oncol.* **2024**, *14*, 1350132. https://doi.org/10.3389/fonc.2024.1273437.
104. Sharma, D.; Carter, H.; Sannachi, L.; Cui, W.; Giles, A.; Saifuddin, M.; Czarnota, G.J. Quantitative ultrasound for evaluation of tumour response to ultrasound-microbubbles and hyperthermia. *Technol. Cancer Res. Treat.* **2023**, *22*, 15330338231200993. https://doi.org/10.1177/15330338231200993.
106. Kolios, M.C.; Czarnota, G.J.; Lee, M.; Hunt, J.W.; Sherar, M.D. Ultrasonic spectral parameter characterization of apoptosis. *Ultrasound Med. Biol.* **2002**, *28*, 589–597. https://doi.org/10.1016/S0301-5629(02)00492-1.
108. Sannachi, L.; Gangeh, M.; Naini, A.S.; Bhargava, P.; Jain, A.; Tran, W.T.; Czarnota, G.J. Quantitative ultrasound monitoring of breast tumour response to neoadjuvant chemotherapy: Comparison of results among clinical scanners. *Ultrasound Med. Biol.* **2020**, *46*, 1142–1157. https://doi.org/10.1016/j.ultrasmedbio.2020.01.022.
109. Piotrzkowska-Wróblewska, H.; Dobruch-Sobczak, K.; Klimonda, Z.; Karwat, P.; Roszkowska-Purska, K.; Gumowska, M.; Litniewski, J. Monitoring breast cancer response to neoadjuvant chemotherapy with ultrasound signal statistics and integrated backscatter. *PLoS ONE* **2019**, *14*, e0213749. https://doi.org/10.1371/journal.pone.0213749.
110. Klimonda, Z.; Karwat, P.; Dobruch-Sobczak, K.; Piotrzkowska-Wróblewska, H.; Litniewski, J. Assessment of breast cancer response to neoadjuvant chemotherapy based on ultrasound backscattering envelope statistics. *Med. Phys.* **2022**, *49*, 1047–1054. https://doi.org/10.1002/mp.15428.
111. Moore-Palhares, D.; Sannachi, L.; Chan, A.W.; Dasgupta, A.; DiCenzo, D.; Gandhi, S.; Pezo, R.; Eisen, A.; Warner, E.; Wright, F.; et al. Validation of a quantitative ultrasound texture analysis model for early prediction of neoadjuvant chemotherapy response in breast cancer: A prospective serial imaging study. *Cancers* **2025**, *17*, 2594. https://doi.org/10.3390/cancers17152594.
112. Use of Quantitative Ultrasound to Guide Adaptive Chemotherapy Among Women with Breast Cancer. ClinicalTrials.gov Identifier:NCT04050228. Available online: https://clinicaltrials.gov/study/NCT04050228 (accessed on 14 October 2025).
114. Mao, N.; Dai, Y.; Zhou, H.; Lin, F.; Zheng, T.; Li, Z.; Yang, P.; Zhao, F.; Li, Q.; Wang, W.; et al. A multimodal and fully automated system for prediction of pathological complete response to neoadjuvant chemotherapy in breast cancer. *Sci. Adv.* **2025**, *11*, eadr1576. https://doi.org/10.1126/sciadv.adr1576.
119. Huang, J.X.; Wu, L.; Wang, X.Y.; Lin, S.Y.; Xu, Y.F.; Wei, M.J.; Pei, X.Q. Delta radiomics based on longitudinal dual-modal ultrasound can early predict response to neoadjuvant chemotherapy in breast cancer patients. *Acad. Radiol.* **2024**, *31*, 1738–1747. https://doi.org/10.1016/j.acra.2023.10.051.
**Missing Acknowledgments**

An Acknowledgments section has been added to the original publication to disclose the use of AI. The authors state that AI tools (GPT-5.6 Thinking, OpenAI) were used solely to assist with formatting references according to the journal style. No AI tools were used to write, edit, or generate the scientific content of the manuscript.

The authors state that the scientific conclusions are unaffected. This correction was approved by the Academic Editor. The original publication has also been updated.
